# Measurement of prompt and nonprompt charmonium suppression in $\text {PbPb}$ collisions at 5.02$\,\text {Te}\text {V}$

**DOI:** 10.1140/epjc/s10052-018-5950-6

**Published:** 2018-06-20

**Authors:** A. M. Sirunyan, A. Tumasyan, W. Adam, F. Ambrogi, E. Asilar, T. Bergauer, J. Brandstetter, E. Brondolin, M. Dragicevic, J. Erö, A. Escalante Del Valle, M. Flechl, M. Friedl, R. Frühwirth, V. M. Ghete, J. Grossmann, J. Hrubec, M. Jeitler, A. König, N. Krammer, I. Krätschmer, D. Liko, T. Madlener, I. Mikulec, E. Pree, N. Rad, H. Rohringer, J. Schieck, R. Schöfbeck, M. Spanring, D. Spitzbart, W. Waltenberger, J. Wittmann, C.-E. Wulz, M. Zarucki, V. Chekhovsky, V. Mossolov, J. Suarez Gonzalez, E. A. De Wolf, D. Di Croce, X. Janssen, J. Lauwers, M. Van De Klundert, H. Van Haevermaet, P. Van Mechelen, N. Van Remortel, S. Abu Zeid, F. Blekman, J. D’Hondt, I. De Bruyn, J. De Clercq, K. Deroover, G. Flouris, D. Lontkovskyi, S. Lowette, I. Marchesini, S. Moortgat, L. Moreels, Q. Python, K. Skovpen, S. Tavernier, W. Van Doninck, P. Van Mulders, I. Van Parijs, D. Beghin, B. Bilin, H. Brun, B. Clerbaux, G. De Lentdecker, H. Delannoy, B. Dorney, G. Fasanella, L. Favart, R. Goldouzian, A. Grebenyuk, A. K. Kalsi, T. Lenzi, J. Luetic, T. Maerschalk, A. Marinov, T. Seva, E. Starling, C. Vander Velde, P. Vanlaer, D. Vannerom, R. Yonamine, F. Zenoni, T. Cornelis, D. Dobur, A. Fagot, M. Gul, I. Khvastunov, D. Poyraz, C. Roskas, S. Salva, M. Tytgat, W. Verbeke, N. Zaganidis, H. Bakhshiansohi, O. Bondu, S. Brochet, G. Bruno, C. Caputo, A. Caudron, P. David, S. De Visscher, C. Delaere, M. Delcourt, B. Francois, A. Giammanco, M. Komm, G. Krintiras, V. Lemaitre, A. Magitteri, A. Mertens, M. Musich, K. Piotrzkowski, L. Quertenmont, A. Saggio, M. Vidal Marono, S. Wertz, J. Zobec, W. L. Aldá Júnior, F. L. Alves, G. A. Alves, L. Brito, M. Correa Martins Junior, C. Hensel, A. Moraes, M. E. Pol, P. Rebello Teles, E. Belchior Batista Das Chagas, W. Carvalho, J. Chinellato, E. Coelho, E. M. Da Costa, G. G. Da Silveira, D. De Jesus Damiao, S. Fonseca De Souza, L. M. Huertas Guativa, H. Malbouisson, M. Melo De Almeida, C. Mora Herrera, L. Mundim, H. Nogima, L. J. Sanchez Rosas, A. Santoro, A. Sznajder, M. Thiel, E. J. Tonelli Manganote, F. Torres Da Silva De Araujo, A. Vilela Pereira, S. Ahuja, C. A. Bernardes, T. R. Fernandez Perez Tomei, E. M. Gregores, P. G. Mercadante, S. F. Novaes, Sandra S. Padula, D. Romero Abad, J. C. Ruiz Vargas, A. Aleksandrov, R. Hadjiiska, P. Iaydjiev, M. Misheva, M. Rodozov, M. Shopova, G. Sultanov, A. Dimitrov, L. Litov, B. Pavlov, P. Petkov, W. Fang, X. Gao, L. Yuan, M. Ahmad, J. G. Bian, G. M. Chen, H. S. Chen, M. Chen, Y. Chen, C. H. Jiang, D. Leggat, H. Liao, Z. Liu, F. Romeo, S. M. Shaheen, A. Spiezia, J. Tao, C. Wang, Z. Wang, E. Yazgan, H. Zhang, S. Zhang, J. Zhao, Y. Ban, G. Chen, J. Li, Q. Li, S. Liu, Y. Mao, S. J. Qian, D. Wang, Z. Xu, F. Zhang, Y. Wang, C. Avila, A. Cabrera, C. A. Carrillo Montoya, L. F. Chaparro Sierra, C. Florez, C. F. González Hernández, J. D. Ruiz Alvarez, M. A. Segura Delgado, B. Courbon, N. Godinovic, D. Lelas, I. Puljak, P. M. Ribeiro Cipriano, T. Sculac, Z. Antunovic, M. Kovac, V. Brigljevic, D. Ferencek, K. Kadija, B. Mesic, A. Starodumov, T. Susa, M. W. Ather, A. Attikis, G. Mavromanolakis, J. Mousa, C. Nicolaou, F. Ptochos, P. A. Razis, H. Rykaczewski, M. Finger, M. Finger, E. Carrera Jarrin, M. A. Mahmoud, Y. Mohammed, E. Salama, R. K. Dewanjee, M. Kadastik, L. Perrini, M. Raidal, A. Tiko, C. Veelken, P. Eerola, H. Kirschenmann, J. Pekkanen, M. Voutilainen, J. Havukainen, J. K. Heikkilä, T. Järvinen, V. Karimäki, R. Kinnunen, T. Lampén, K. Lassila-Perini, S. Laurila, S. Lehti, T. Lindén, P. Luukka, H. Siikonen, E. Tuominen, J. Tuominiemi, T. Tuuva, M. Besancon, F. Couderc, M. Dejardin, D. Denegri, J. L. Faure, F. Ferri, S. Ganjour, S. Ghosh, P. Gras, G. Hamel de Monchenault, P. Jarry, I. Kucher, C. Leloup, E. Locci, M. Machet, J. Malcles, G. Negro, J. Rander, A. Rosowsky, M. Ö. Sahin, M. Titov, A. Abdulsalam, C. Amendola, I. Antropov, S. Baffioni, F. Beaudette, P. Busson, L. Cadamuro, C. Charlot, R. Granier de Cassagnac, M. Jo, S. Lisniak, A. Lobanov, J. Martin Blanco, M. Nguyen, C. Ochando, G. Ortona, P. Paganini, P. Pigard, R. Salerno, J. B. Sauvan, Y. Sirois, A. G. Stahl Leiton, T. Strebler, Y. Yilmaz, A. Zabi, A. Zghiche, J.-L. Agram, J. Andrea, D. Bloch, J.-M. Brom, M. Buttignol, E. C. Chabert, N. Chanon, C. Collard, E. Conte, X. Coubez, J.-C. Fontaine, D. Gelé, U. Goerlach, M. Jansová, A.-C. Le Bihan, N. Tonon, P. Van Hove, S. Gadrat, S. Beauceron, C. Bernet, G. Boudoul, R. Chierici, D. Contardo, P. Depasse, H. El Mamouni, J. Fay, L. Finco, S. Gascon, M. Gouzevitch, G. Grenier, B. Ille, F. Lagarde, I. B. Laktineh, M. Lethuillier, L. Mirabito, A. L. Pequegnot, S. Perries, A. Popov, V. Sordini, M. Vander Donckt, S. Viret, T. Toriashvili, Z. Tsamalaidze, C. Autermann, L. Feld, M. K. Kiesel, K. Klein, M. Lipinski, M. Preuten, C. Schomakers, J. Schulz, M. Teroerde, V. Zhukov, A. Albert, E. Dietz-Laursonn, D. Duchardt, M. Endres, M. Erdmann, S. Erdweg, T. Esch, R. Fischer, A. Güth, M. Hamer, T. Hebbeker, C. Heidemann, K. Hoepfner, S. Knutzen, M. Merschmeyer, A. Meyer, P. Millet, S. Mukherjee, T. Pook, M. Radziej, H. Reithler, M. Rieger, F. Scheuch, D. Teyssier, S. Thüer, G. Flügge, B. Kargoll, T. Kress, A. Künsken, T. Müller, A. Nehrkorn, A. Nowack, C. Pistone, O. Pooth, A. Stahl, M. Aldaya Martin, T. Arndt, C. Asawatangtrakuldee, K. Beernaert, O. Behnke, U. Behrens, A. Bermúdez Martínez, A. A. Bin Anuar, K. Borras, V. Botta, A. Campbell, P. Connor, C. Contreras-Campana, F. Costanza, C. Diez Pardos, G. Eckerlin, D. Eckstein, T. Eichhorn, E. Eren, E. Gallo, J. Garay Garcia, A. Geiser, J. M. Grados Luyando, A. Grohsjean, P. Gunnellini, M. Guthoff, A. Harb, J. Hauk, M. Hempel, H. Jung, M. Kasemann, J. Keaveney, C. Kleinwort, I. Korol, D. Krücker, W. Lange, A. Lelek, T. Lenz, J. Leonard, K. Lipka, W. Lohmann, R. Mankel, I.-A. Melzer-Pellmann, A. B. Meyer, G. Mittag, J. Mnich, A. Mussgiller, E. Ntomari, D. Pitzl, A. Raspereza, M. Savitskyi, P. Saxena, R. Shevchenko, N. Stefaniuk, G. P. Van Onsem, R. Walsh, Y. Wen, K. Wichmann, C. Wissing, O. Zenaiev, R. Aggleton, S. Bein, V. Blobel, M. Centis Vignali, T. Dreyer, E. Garutti, D. Gonzalez, J. Haller, A. Hinzmann, M. Hoffmann, A. Karavdina, R. Klanner, R. Kogler, N. Kovalchuk, S. Kurz, T. Lapsien, D. Marconi, M. Meyer, M. Niedziela, D. Nowatschin, F. Pantaleo, T. Peiffer, A. Perieanu, C. Scharf, P. Schleper, A. Schmidt, S. Schumann, J. Schwandt, J. Sonneveld, H. Stadie, G. Steinbrück, F. M. Stober, M. Stöver, H. Tholen, D. Troendle, E. Usai, A. Vanhoefer, B. Vormwald, M. Akbiyik, C. Barth, M. Baselga, S. Baur, E. Butz, R. Caspart, T. Chwalek, F. Colombo, W. De Boer, A. Dierlamm, N. Faltermann, B. Freund, R. Friese, M. Giffels, M. A. Harrendorf, F. Hartmann, S. M. Heindl, U. Husemann, F. Kassel, S. Kudella, H. Mildner, M. U. Mozer, Th. Müller, M. Plagge, G. Quast, K. Rabbertz, M. Schröder, I. Shvetsov, G. Sieber, H. J. Simonis, R. Ulrich, S. Wayand, M. Weber, T. Weiler, S. Williamson, C. Wöhrmann, R. Wolf, G. Anagnostou, G. Daskalakis, T. Geralis, A. Kyriakis, D. Loukas, I. Topsis-Giotis, G. Karathanasis, S. Kesisoglou, A. Panagiotou, N. Saoulidou, K. Kousouris, I. Evangelou, C. Foudas, P. Gianneios, P. Katsoulis, P. Kokkas, S. Mallios, N. Manthos, I. Papadopoulos, E. Paradas, J. Strologas, F. A. Triantis, D. Tsitsonis, M. Csanad, N. Filipovic, G. Pasztor, O. Surányi, G. I. Veres, G. Bencze, C. Hajdu, D. Horvath, Á. Hunyadi, F. Sikler, V. Veszpremi, N. Beni, S. Czellar, J. Karancsi, A. Makovec, J. Molnar, Z. Szillasi, M. Bartók, P. Raics, Z. L. Trocsanyi, B. Ujvari, S. Choudhury, J. R. Komaragiri, S. Bahinipati, S. Bhowmik, P. Mal, K. Mandal, A. Nayak, D. K. Sahoo, N. Sahoo, S. K. Swain, S. Bansal, S. B. Beri, V. Bhatnagar, R. Chawla, N. Dhingra, A. Kaur, M. Kaur, S. Kaur, R. Kumar, P. Kumari, A. Mehta, J. B. Singh, G. Walia, Ashok Kumar, Aashaq Shah, A. Bhardwaj, S. Chauhan, B. C. Choudhary, R. B. Garg, S. Keshri, A. Kumar, S. Malhotra, M. Naimuddin, K. Ranjan, R. Sharma, R. Bhardwaj, R. Bhattacharya, S. Bhattacharya, U. Bhawandeep, S. Dey, S. Dutt, S. Dutta, S. Ghosh, N. Majumdar, A. Modak, K. Mondal, S. Mukhopadhyay, S. Nandan, A. Purohit, A. Roy, S. Roy Chowdhury, S. Sarkar, M. Sharan, S. Thakur, P. K. Behera, R. Chudasama, D. Dutta, V. Jha, V. Kumar, A. K. Mohanty, P. K. Netrakanti, L. M. Pant, P. Shukla, A. Topkar, T. Aziz, S. Dugad, B. Mahakud, S. Mitra, G. B. Mohanty, N. Sur, B. Sutar, S. Banerjee, S. Bhattacharya, S. Chatterjee, P. Das, M. Guchait, Sa. Jain, S. Kumar, M. Maity, G. Majumder, K. Mazumdar, T. Sarkar, N. Wickramage, S. Chauhan, S. Dube, V. Hegde, A. Kapoor, K. Kothekar, S. Pandey, A. Rane, S. Sharma, S. Chenarani, E. Eskandari Tadavani, S. M. Etesami, M. Khakzad, M. Mohammadi Najafabadi, M. Naseri, S. Paktinat Mehdiabadi, F. Rezaei Hosseinabadi, B. Safarzadeh, M. Zeinali, M. Felcini, M. Grunewald, M. Abbrescia, C. Calabria, A. Colaleo, D. Creanza, L. Cristella, N. De Filippis, M. De Palma, F. Errico, L. Fiore, G. Iaselli, S. Lezki, G. Maggi, M. Maggi, G. Miniello, S. My, S. Nuzzo, A. Pompili, G. Pugliese, R. Radogna, A. Ranieri, G. Selvaggi, A. Sharma, L. Silvestris, R. Venditti, P. Verwilligen, G. Abbiendi, C. Battilana, D. Bonacorsi, L. Borgonovi, S. Braibant-Giacomelli, R. Campanini, P. Capiluppi, A. Castro, F. R. Cavallo, S. S. Chhibra, G. Codispoti, M. Cuffiani, G. M. Dallavalle, F. Fabbri, A. Fanfani, D. Fasanella, P. Giacomelli, C. Grandi, L. Guiducci, S. Marcellini, G. Masetti, A. Montanari, F. L. Navarria, A. Perrotta, A. M. Rossi, T. Rovelli, G. P. Siroli, N. Tosi, S. Albergo, S. Costa, A. Di Mattia, F. Giordano, R. Potenza, A. Tricomi, C. Tuve, G. Barbagli, K. Chatterjee, V. Ciulli, C. Civinini, R. D’Alessandro, E. Focardi, P. Lenzi, M. Meschini, S. Paoletti, L. Russo, G. Sguazzoni, D. Strom, L. Viliani, L. Benussi, S. Bianco, F. Fabbri, D. Piccolo, F. Primavera, V. Calvelli, F. Ferro, F. Ravera, E. Robutti, S. Tosi, A. Benaglia, A. Beschi, L. Brianza, F. Brivio, V. Ciriolo, M. E. Dinardo, S. Fiorendi, S. Gennai, A. Ghezzi, P. Govoni, M. Malberti, S. Malvezzi, R. A. Manzoni, D. Menasce, L. Moroni, M. Paganoni, K. Pauwels, D. Pedrini, S. Pigazzini, S. Ragazzi, T. Tabarelli de Fatis, S. Buontempo, N. Cavallo, S. Di Guida, F. Fabozzi, F. Fienga, A. O. M. Iorio, W. A. Khan, L. Lista, S. Meola, P. Paolucci, C. Sciacca, F. Thyssen, P. Azzi, N. Bacchetta, L. Benato, D. Bisello, A. Boletti, R. Carlin, A. Carvalho Antunes De Oliveira, P. Checchia, M. Dall’Osso, P. De Castro Manzano, T. Dorigo, F. Gasparini, U. Gasparini, A. Gozzelino, S. Lacaprara, P. Lujan, M. Margoni, A. T. Meneguzzo, N. Pozzobon, P. Ronchese, R. Rossin, F. Simonetto, E. Torassa, S. Ventura, M. Zanetti, P. Zotto, A. Braghieri, A. Magnani, P. Montagna, S. P. Ratti, V. Re, M. Ressegotti, C. Riccardi, P. Salvini, I. Vai, P. Vitulo, L. Alunni Solestizi, M. Biasini, G. M. Bilei, C. Cecchi, D. Ciangottini, L. Fanò, R. Leonardi, E. Manoni, G. Mantovani, V. Mariani, M. Menichelli, A. Rossi, A. Santocchia, D. Spiga, K. Androsov, P. Azzurri, G. Bagliesi, T. Boccali, L. Borrello, R. Castaldi, M. A. Ciocci, R. Dell’Orso, G. Fedi, L. Giannini, A. Giassi, M. T. Grippo, F. Ligabue, T. Lomtadze, E. Manca, G. Mandorli, A. Messineo, F. Palla, A. Rizzi, A. Savoy-Navarro, P. Spagnolo, R. Tenchini, G. Tonelli, A. Venturi, P. G. Verdini, L. Barone, F. Cavallari, M. Cipriani, N. Daci, D. Del Re, E. Di Marco, M. Diemoz, S. Gelli, E. Longo, F. Margaroli, B. Marzocchi, P. Meridiani, G. Organtini, R. Paramatti, F. Preiato, S. Rahatlou, C. Rovelli, F. Santanastasio, N. Amapane, R. Arcidiacono, S. Argiro, M. Arneodo, N. Bartosik, R. Bellan, C. Biino, N. Cartiglia, F. Cenna, M. Costa, R. Covarelli, A. Degano, N. Demaria, B. Kiani, C. Mariotti, S. Maselli, E. Migliore, V. Monaco, E. Monteil, M. Monteno, M. M. Obertino, L. Pacher, N. Pastrone, M. Pelliccioni, G. L. Pinna Angioni, A. Romero, M. Ruspa, R. Sacchi, K. Shchelina, V. Sola, A. Solano, A. Staiano, P. Traczyk, S. Belforte, M. Casarsa, F. Cossutti, G. Della Ricca, A. Zanetti, D. H. Kim, G. N. Kim, M. S. Kim, J. Lee, S. Lee, S. W. Lee, C. S. Moon, Y. D. Oh, S. Sekmen, D. C. Son, Y. C. Yang, A. Lee, H. Kim, D. H. Moon, G. Oh, J. A. Brochero Cifuentes, J. Goh, T. J. Kim, S. Cho, S. Choi, Y. Go, D. Gyun, S. Ha, B. Hong, Y. Jo, Y. Kim, K. Lee, K. S. Lee, S. Lee, J. Lim, S. K. Park, Y. Roh, J. Almond, J. Kim, J. S. Kim, H. Lee, K. Lee, K. Nam, S. B. Oh, B. C. Radburn-Smith, S. h. Seo, U. K. Yang, H. D. Yoo, G. B. Yu, H. Kim, J. H. Kim, J. S. H. Lee, I. C. Park, Y. Choi, C. Hwang, J. Lee, I. Yu, V. Dudenas, A. Juodagalvis, J. Vaitkus, I. Ahmed, Z. A. Ibrahim, M. A. B. Md Ali, F. Mohamad Idris, W. A. T. Wan Abdullah, M. N. Yusli, Z. Zolkapli, R. Reyes-Almanza, G. Ramirez-Sanchez, M. C. Duran-Osuna, H. Castilla-Valdez, E. De La Cruz-Burelo, I. Heredia-De La Cruz, R. I. Rabadan-Trejo, R. Lopez-Fernandez, J. Mejia Guisao, A. Sanchez-Hernandez, S. Carrillo Moreno, C. Oropeza Barrera, F. Vazquez Valencia, J. Eysermans, I. Pedraza, H. A. Salazar Ibarguen, C. Uribe Estrada, A. Morelos Pineda, D. Krofcheck, P. H. Butler, A. Ahmad, M. Ahmad, Q. Hassan, H. R. Hoorani, A. Saddique, M. A. Shah, M. Shoaib, M. Waqas, H. Bialkowska, M. Bluj, B. Boimska, T. Frueboes, M. Górski, M. Kazana, K. Nawrocki, M. Szleper, P. Zalewski, K. Bunkowski, A. Byszuk, K. Doroba, A. Kalinowski, M. Konecki, J. Krolikowski, M. Misiura, M. Olszewski, A. Pyskir, M. Walczak, P. Bargassa, C. Beirão Da Cruz E Silva, A. Di Francesco, P. Faccioli, B. Galinhas, M. Gallinaro, J. Hollar, N. Leonardo, L. Lloret Iglesias, M. V. Nemallapudi, J. Seixas, G. Strong, O. Toldaiev, D. Vadruccio, J. Varela, A. Baginyan, A. Golunov, I. Golutvin, V. Karjavin, V. Korenkov, G. Kozlov, A. Lanev, A. Malakhov, V. Matveev, V. V. Mitsyn, V. Palichik, V. Perelygin, S. Shmatov, N. Skatchkov, V. Smirnov, B. S. Yuldashev, A. Zarubin, V. Zhiltsov, Y. Ivanov, V. Kim, E. Kuznetsova, P. Levchenko, V. Murzin, V. Oreshkin, I. Smirnov, D. Sosnov, V. Sulimov, L. Uvarov, S. Vavilov, A. Vorobyev, Yu. Andreev, A. Dermenev, S. Gninenko, N. Golubev, A. Karneyeu, M. Kirsanov, N. Krasnikov, A. Pashenkov, D. Tlisov, A. Toropin, V. Epshteyn, V. Gavrilov, N. Lychkovskaya, V. Popov, I. Pozdnyakov, G. Safronov, A. Spiridonov, A. Stepennov, M. Toms, E. Vlasov, A. Zhokin, T. Aushev, A. Bylinkin, M. Chadeeva, P. Parygin, D. Philippov, S. Polikarpov, E. Popova, V. Rusinov, V. Andreev, M. Azarkin, I. Dremin, M. Kirakosyan, A. Terkulov, A. Baskakov, A. Belyaev, E. Boos, A. Demiyanov, A. Ershov, A. Gribushin, O. Kodolova, V. Korotkikh, I. Lokhtin, I. Miagkov, S. Obraztsov, S. Petrushanko, V. Savrin, A. Snigirev, I. Vardanyan, V. Blinov, D. Shtol, Y. Skovpen, I. Azhgirey, I. Bayshev, S. Bitioukov, D. Elumakhov, A. Godizov, V. Kachanov, A. Kalinin, D. Konstantinov, P. Mandrik, V. Petrov, R. Ryutin, A. Sobol, S. Troshin, N. Tyurin, A. Uzunian, A. Volkov, P. Adzic, P. Cirkovic, D. Devetak, M. Dordevic, J. Milosevic, V. Rekovic, J. Alcaraz Maestre, I. Bachiller, M. Barrio Luna, M. Cerrada, N. Colino, B. De La Cruz, A. Delgado Peris, C. Fernandez Bedoya, J. P. Fernández Ramos, J. Flix, M. C. Fouz, O. Gonzalez Lopez, S. Goy Lopez, J. M. Hernandez, M. I. Josa, D. Moran, A. Pérez-Calero Yzquierdo, J. Puerta Pelayo, A. Quintario Olmeda, I. Redondo, L. Romero, M. S. Soares, A. Álvarez Fernández, C. Albajar, J. F. de Trocóniz, M. Missiroli, J. Cuevas, C. Erice, J. Fernandez Menendez, I. Gonzalez Caballero, J. R. González Fernández, E. Palencia Cortezon, S. Sanchez Cruz, P. Vischia, J. M. Vizan Garcia, I. J. Cabrillo, A. Calderon, B. Chazin Quero, E. Curras, J. Duarte Campderros, M. Fernandez, J. Garcia-Ferrero, G. Gomez, A. Lopez Virto, J. Marco, C. Martinez Rivero, P. Martinez Ruiz del Arbol, F. Matorras, J. Piedra Gomez, T. Rodrigo, A. Ruiz-Jimeno, L. Scodellaro, N. Trevisani, I. Vila, R. Vilar Cortabitarte, D. Abbaneo, B. Akgun, E. Auffray, P. Baillon, A. H. Ball, D. Barney, J. Bendavid, M. Bianco, P. Bloch, A. Bocci, C. Botta, T. Camporesi, R. Castello, M. Cepeda, G. Cerminara, E. Chapon, Y. Chen, D. d’Enterria, A. Dabrowski, V. Daponte, A. David, M. De Gruttola, A. De Roeck, N. Deelen, M. Dobson, T. du Pree, M. Dünser, N. Dupont, A. Elliott-Peisert, P. Everaerts, F. Fallavollita, G. Franzoni, J. Fulcher, W. Funk, D. Gigi, A. Gilbert, K. Gill, F. Glege, D. Gulhan, P. Harris, J. Hegeman, V. Innocente, A. Jafari, P. Janot, O. Karacheban, J. Kieseler, V. Knünz, A. Kornmayer, M. J. Kortelainen, M. Krammer, C. Lange, P. Lecoq, C. Lourenço, M. T. Lucchini, L. Malgeri, M. Mannelli, A. Martelli, F. Meijers, J. A. Merlin, S. Mersi, E. Meschi, P. Milenovic, F. Moortgat, M. Mulders, H. Neugebauer, J. Ngadiuba, S. Orfanelli, L. Orsini, L. Pape, E. Perez, M. Peruzzi, A. Petrilli, G. Petrucciani, A. Pfeiffer, M. Pierini, D. Rabady, A. Racz, T. Reis, G. Rolandi, M. Rovere, H. Sakulin, C. Schäfer, C. Schwick, M. Seidel, M. Selvaggi, A. Sharma, P. Silva, P. Sphicas, A. Stakia, J. Steggemann, M. Stoye, M. Tosi, D. Treille, A. Triossi, A. Tsirou, V. Veckalns, M. Verweij, W. D. Zeuner, W. Bertl, L. Caminada, K. Deiters, W. Erdmann, R. Horisberger, Q. Ingram, H. C. Kaestli, D. Kotlinski, U. Langenegger, T. Rohe, S. A. Wiederkehr, M. Backhaus, L. Bäni, P. Berger, L. Bianchini, B. Casal, G. Dissertori, M. Dittmar, M. Donegà, C. Dorfer, C. Grab, C. Heidegger, D. Hits, J. Hoss, G. Kasieczka, T. Klijnsma, W. Lustermann, B. Mangano, M. Marionneau, M. T. Meinhard, D. Meister, F. Micheli, P. Musella, F. Nessi-Tedaldi, F. Pandolfi, J. Pata, F. Pauss, G. Perrin, L. Perrozzi, M. Quittnat, M. Reichmann, D. A. Sanz Becerra, M. Schönenberger, L. Shchutska, V. R. Tavolaro, K. Theofilatos, M. L. Vesterbacka Olsson, R. Wallny, D. H. Zhu, T. K. Aarrestad, C. Amsler, M. F. Canelli, A. De Cosa, R. Del Burgo, S. Donato, C. Galloni, T. Hreus, B. Kilminster, D. Pinna, G. Rauco, P. Robmann, D. Salerno, K. Schweiger, C. Seitz, Y. Takahashi, A. Zucchetta, V. Candelise, Y. H. Chang, K. y. Cheng, T. H. Doan, Sh. Jain, R. Khurana, C. M. Kuo, W. Lin, A. Pozdnyakov, S. S. Yu, Arun Kumar, P. Chang, Y. Chao, K. F. Chen, P. H. Chen, F. Fiori, W.-S. Hou, Y. Hsiung, Y. F. Liu, R.-S. Lu, E. Paganis, A. Psallidas, A. Steen, J. f. Tsai, B. Asavapibhop, K. Kovitanggoon, G. Singh, N. Srimanobhas, M. N. Bakirci, A. Bat, F. Boran, S. Damarseckin, Z. S. Demiroglu, C. Dozen, E. Eskut, S. Girgis, G. Gokbulut, Y. Guler, I. Hos, E. E. Kangal, O. Kara, U. Kiminsu, M. Oglakci, G. Onengut, K. Ozdemir, S. Ozturk, D. Sunar Cerci, U. G. Tok, H. Topakli, S. Turkcapar, I. S. Zorbakir, C. Zorbilmez, G. Karapinar, K. Ocalan, M. Yalvac, M. Zeyrek, E. Gülmez, M. Kaya, O. Kaya, S. Tekten, E. A. Yetkin, M. N. Agaras, S. Atay, A. Cakir, K. Cankocak, I. Köseoglu, B. Grynyov, L. Levchuk, F. Ball, L. Beck, J. J. Brooke, D. Burns, E. Clement, D. Cussans, O. Davignon, H. Flacher, J. Goldstein, G. P. Heath, H. F. Heath, L. Kreczko, D. M. Newbold, S. Paramesvaran, T. Sakuma, S. Seif El Nasr-storey, D. Smith, V. J. Smith, A. Belyaev, C. Brew, R. M. Brown, L. Calligaris, D. Cieri, D. J. A. Cockerill, J. A. Coughlan, K. Harder, S. Harper, J. Linacre, E. Olaiya, D. Petyt, C. H. Shepherd-Themistocleous, A. Thea, I. R. Tomalin, T. Williams, G. Auzinger, R. Bainbridge, J. Borg, S. Breeze, O. Buchmuller, A. Bundock, S. Casasso, M. Citron, D. Colling, L. Corpe, P. Dauncey, G. Davies, A. De Wit, M. Della Negra, R. Di Maria, A. Elwood, Y. Haddad, G. Hall, G. Iles, T. James, R. Lane, C. Laner, L. Lyons, A.-M. Magnan, S. Malik, L. Mastrolorenzo, T. Matsushita, J. Nash, A. Nikitenko, V. Palladino, M. Pesaresi, D. M. Raymond, A. Richards, A. Rose, E. Scott, C. Seez, A. Shtipliyski, S. Summers, A. Tapper, K. Uchida, M. Vazquez Acosta, T. Virdee, N. Wardle, D. Winterbottom, J. Wright, S. C. Zenz, J. E. Cole, P. R. Hobson, A. Khan, P. Kyberd, I. D. Reid, L. Teodorescu, S. Zahid, A. Borzou, K. Call, J. Dittmann, K. Hatakeyama, H. Liu, N. Pastika, C. Smith, R. Bartek, A. Dominguez, A. Buccilli, S. I. Cooper, C. Henderson, P. Rumerio, C. West, D. Arcaro, A. Avetisyan, T. Bose, D. Gastler, D. Rankin, C. Richardson, J. Rohlf, L. Sulak, D. Zou, G. Benelli, D. Cutts, A. Garabedian, M. Hadley, J. Hakala, U. Heintz, J. M. Hogan, K. H. M. Kwok, E. Laird, G. Landsberg, J. Lee, Z. Mao, M. Narain, J. Pazzini, S. Piperov, S. Sagir, R. Syarif, D. Yu, R. Band, C. Brainerd, R. Breedon, D. Burns, M. Calderon De La Barca Sanchez, M. Chertok, J. Conway, R. Conway, P. T. Cox, R. Erbacher, C. Flores, G. Funk, W. Ko, R. Lander, C. Mclean, M. Mulhearn, D. Pellett, J. Pilot, S. Shalhout, M. Shi, J. Smith, D. Stolp, K. Tos, M. Tripathi, Z. Wang, M. Bachtis, C. Bravo, R. Cousins, A. Dasgupta, A. Florent, J. Hauser, M. Ignatenko, N. Mccoll, S. Regnard, D. Saltzberg, C. Schnaible, V. Valuev, E. Bouvier, K. Burt, R. Clare, J. Ellison, J. W. Gary, S. M. A. GhiasiShirazi, G. Hanson, J. Heilman, G. Karapostoli, E. Kennedy, F. Lacroix, O. R. Long, M. Olmedo Negrete, M. I. Paneva, W. Si, L. Wang, H. Wei, S. Wimpenny, B. R. Yates, J. G. Branson, S. Cittolin, M. Derdzinski, R. Gerosa, D. Gilbert, B. Hashemi, A. Holzner, D. Klein, G. Kole, V. Krutelyov, J. Letts, M. Masciovecchio, D. Olivito, S. Padhi, M. Pieri, M. Sani, V. Sharma, M. Tadel, A. Vartak, S. Wasserbaech, J. Wood, F. Würthwein, A. Yagil, G. Zevi Della Porta, N. Amin, R. Bhandari, J. Bradmiller-Feld, C. Campagnari, A. Dishaw, V. Dutta, M. Franco Sevilla, L. Gouskos, R. Heller, J. Incandela, A. Ovcharova, H. Qu, J. Richman, D. Stuart, I. Suarez, J. Yoo, D. Anderson, A. Bornheim, J. M. Lawhorn, H. B. Newman, T. Nguyen, C. Pena, M. Spiropulu, J. R. Vlimant, S. Xie, Z. Zhang, R. Y. Zhu, M. B. Andrews, T. Ferguson, T. Mudholkar, M. Paulini, J. Russ, M. Sun, H. Vogel, I. Vorobiev, M. Weinberg, J. P. Cumalat, W. T. Ford, F. Jensen, A. Johnson, M. Krohn, S. Leontsinis, T. Mulholland, K. Stenson, S. R. Wagner, J. Alexander, J. Chaves, J. Chu, S. Dittmer, K. Mcdermott, N. Mirman, J. R. Patterson, D. Quach, A. Rinkevicius, A. Ryd, L. Skinnari, L. Soffi, S. M. Tan, Z. Tao, J. Thom, J. Tucker, P. Wittich, M. Zientek, S. Abdullin, M. Albrow, M. Alyari, G. Apollinari, A. Apresyan, A. Apyan, S. Banerjee, L. A. T. Bauerdick, A. Beretvas, J. Berryhill, P. C. Bhat, G. Bolla, K. Burkett, J. N. Butler, A. Canepa, G. B. Cerati, H. W. K. Cheung, F. Chlebana, M. Cremonesi, J. Duarte, V. D. Elvira, J. Freeman, Z. Gecse, E. Gottschalk, L. Gray, D. Green, S. Grünendahl, O. Gutsche, R. M. Harris, S. Hasegawa, J. Hirschauer, Z. Hu, B. Jayatilaka, S. Jindariani, M. Johnson, U. Joshi, B. Klima, B. Kreis, S. Lammel, D. Lincoln, R. Lipton, M. Liu, T. Liu, R. Lopes De Sá, J. Lykken, K. Maeshima, N. Magini, J. M. Marraffino, D. Mason, P. McBride, P. Merkel, S. Mrenna, S. Nahn, V. O’Dell, K. Pedro, O. Prokofyev, G. Rakness, L. Ristori, B. Schneider, E. Sexton-Kennedy, A. Soha, W. J. Spalding, L. Spiegel, S. Stoynev, J. Strait, N. Strobbe, L. Taylor, S. Tkaczyk, N. V. Tran, L. Uplegger, E. W. Vaandering, C. Vernieri, M. Verzocchi, R. Vidal, M. Wang, H. A. Weber, A. Whitbeck, D. Acosta, P. Avery, P. Bortignon, D. Bourilkov, A. Brinkerhoff, A. Carnes, M. Carver, D. Curry, R. D. Field, I. K. Furic, S. V. Gleyzer, B. M. Joshi, J. Konigsberg, A. Korytov, K. Kotov, P. Ma, K. Matchev, H. Mei, G. Mitselmakher, K. Shi, D. Sperka, N. Terentyev, L. Thomas, J. Wang, S. Wang, J. Yelton, Y. R. Joshi, S. Linn, P. Markowitz, J. L. Rodriguez, A. Ackert, T. Adams, A. Askew, S. Hagopian, V. Hagopian, K. F. Johnson, T. Kolberg, G. Martinez, T. Perry, H. Prosper, A. Saha, A. Santra, V. Sharma, R. Yohay, M. M. Baarmand, V. Bhopatkar, S. Colafranceschi, M. Hohlmann, D. Noonan, T. Roy, F. Yumiceva, M. R. Adams, L. Apanasevich, D. Berry, R. R. Betts, R. Cavanaugh, X. Chen, O. Evdokimov, C. E. Gerber, D. A. Hangal, D. J. Hofman, K. Jung, J. Kamin, I. D. Sandoval Gonzalez, M. B. Tonjes, H. Trauger, N. Varelas, H. Wang, Z. Wu, J. Zhang, B. Bilki, W. Clarida, K. Dilsiz, S. Durgut, R. P. Gandrajula, M. Haytmyradov, V. Khristenko, J.-P. Merlo, H. Mermerkaya, A. Mestvirishvili, A. Moeller, J. Nachtman, H. Ogul, Y. Onel, F. Ozok, A. Penzo, C. Snyder, E. Tiras, J. Wetzel, K. Yi, B. Blumenfeld, A. Cocoros, N. Eminizer, D. Fehling, L. Feng, A. V. Gritsan, P. Maksimovic, J. Roskes, U. Sarica, M. Swartz, M. Xiao, C. You, A. Al-bataineh, P. Baringer, A. Bean, S. Boren, J. Bowen, J. Castle, S. Khalil, A. Kropivnitskaya, D. Majumder, W. Mcbrayer, M. Murray, C. Royon, S. Sanders, E. Schmitz, J. D. Tapia Takaki, Q. Wang, A. Ivanov, K. Kaadze, Y. Maravin, A. Mohammadi, L. K. Saini, N. Skhirtladze, F. Rebassoo, D. Wright, C. Anelli, A. Baden, O. Baron, A. Belloni, S. C. Eno, Y. Feng, C. Ferraioli, N. J. Hadley, S. Jabeen, G. Y. Jeng, R. G. Kellogg, J. Kunkle, A. C. Mignerey, F. Ricci-Tam, Y. H. Shin, A. Skuja, S. C. Tonwar, D. Abercrombie, B. Allen, V. Azzolini, R. Barbieri, A. Baty, R. Bi, S. Brandt, W. Busza, I. A. Cali, M. D’Alfonso, Z. Demiragli, G. Gomez Ceballos, M. Goncharov, D. Hsu, M. Hu, Y. Iiyama, G. M. Innocenti, M. Klute, D. Kovalskyi, Y.-J. Lee, A. Levin, P. D. Luckey, B. Maier, A. C. Marini, C. Mcginn, C. Mironov, S. Narayanan, X. Niu, C. Paus, C. Roland, G. Roland, J. Salfeld-Nebgen, G. S. F. Stephans, K. Tatar, D. Velicanu, J. Wang, T. W. Wang, B. Wyslouch, A. C. Benvenuti, R. M. Chatterjee, A. Evans, P. Hansen, J. Hiltbrand, S. Kalafut, Y. Kubota, Z. Lesko, J. Mans, S. Nourbakhsh, N. Ruckstuhl, R. Rusack, J. Turkewitz, M. A. Wadud, J. G. Acosta, S. Oliveros, E. Avdeeva, K. Bloom, D. R. Claes, C. Fangmeier, F. Golf, R. GonzalezSuarez, R. Kamalieddin, I. Kravchenko, J. Monroy, J. E. Siado, G. R. Snow, B. Stieger, J. Dolen, A. Godshalk, C. Harrington, I. Iashvili, D. Nguyen, A. Parker, S. Rappoccio, B. Roozbahani, G. Alverson, E. Barberis, C. Freer, A. Hortiangtham, A. Massironi, D. M. Morse, T. Orimoto, R. Teixeira De Lima, D. Trocino, T. Wamorkar, B. Wang, A. Wisecarver, D. Wood, S. Bhattacharya, O. Charaf, K. A. Hahn, N. Mucia, N. Odell, M. H. Schmitt, K. Sung, M. Trovato, M. Velasco, R. Bucci, N. Dev, M. Hildreth, K. Hurtado Anampa, C. Jessop, D. J. Karmgard, N. Kellams, K. Lannon, W. Li, N. Loukas, N. Marinelli, F. Meng, C. Mueller, Y. Musienko, M. Planer, A. Reinsvold, R. Ruchti, P. Siddireddy, G. Smith, S. Taroni, M. Wayne, A. Wightman, M. Wolf, A. Woodard, J. Alimena, L. Antonelli, B. Bylsma, L. S. Durkin, S. Flowers, B. Francis, A. Hart, C. Hill, W. Ji, B. Liu, W. Luo, B. L. Winer, H. W. Wulsin, S. Cooperstein, O. Driga, P. Elmer, J. Hardenbrook, P. Hebda, S. Higginbotham, A. Kalogeropoulos, D. Lange, J. Luo, D. Marlow, K. Mei, I. Ojalvo, J. Olsen, C. Palmer, P. Piroué, D. Stickland, C. Tully, S. Malik, S. Norberg, A. Barker, V. E. Barnes, S. Das, S. Folgueras, L. Gutay, M. K. Jha, M. Jones, A. W. Jung, A. Khatiwada, D. H. Miller, N. Neumeister, C. C. Peng, H. Qiu, J. F. Schulte, J. Sun, F. Wang, R. Xiao, W. Xie, T. Cheng, N. Parashar, J. Stupak, Z. Chen, K. M. Ecklund, S. Freed, F. J. M. Geurts, M. Guilbaud, M. Kilpatrick, W. Li, B. Michlin, B. P. Padley, J. Roberts, J. Rorie, W. Shi, Z. Tu, J. Zabel, A. Zhang, A. Bodek, P. de Barbaro, R. Demina, Y. t. Duh, T. Ferbel, M. Galanti, A. Garcia-Bellido, J. Han, O. Hindrichs, A. Khukhunaishvili, K. H. Lo, P. Tan, M. Verzetti, R. Ciesielski, K. Goulianos, C. Mesropian, A. Agapitos, J. P. Chou, Y. Gershtein, T. A. Gómez Espinosa, E. Halkiadakis, M. Heindl, E. Hughes, S. Kaplan, R. Kunnawalkam Elayavalli, S. Kyriacou, A. Lath, R. Montalvo, K. Nash, M. Osherson, H. Saka, S. Salur, S. Schnetzer, D. Sheffield, S. Somalwar, R. Stone, S. Thomas, P. Thomassen, M. Walker, A. G. Delannoy, J. Heideman, G. Riley, K. Rose, S. Spanier, K. Thapa, O. Bouhali, A. Castaneda Hernandez, A. Celik, M. Dalchenko, M. De Mattia, A. Delgado, S. Dildick, R. Eusebi, J. Gilmore, T. Huang, T. Kamon, R. Mueller, Y. Pakhotin, R. Patel, A. Perloff, L. Perniè, D. Rathjens, A. Safonov, A. Tatarinov, K. A. Ulmer, N. Akchurin, J. Damgov, F. De Guio, P. R. Dudero, J. Faulkner, E. Gurpinar, S. Kunori, K. Lamichhane, S. W. Lee, T. Libeiro, T. Mengke, S. Muthumuni, T. Peltola, S. Undleeb, I. Volobouev, Z. Wang, S. Greene, A. Gurrola, R. Janjam, W. Johns, C. Maguire, A. Melo, H. Ni, K. Padeken, P. Sheldon, S. Tuo, J. Velkovska, Q. Xu, M. W. Arenton, P. Barria, B. Cox, R. Hirosky, M. Joyce, A. Ledovskoy, H. Li, C. Neu, T. Sinthuprasith, Y. Wang, E. Wolfe, F. Xia, R. Harr, P. E. Karchin, N. Poudyal, J. Sturdy, P. Thapa, S. Zaleski, M. Brodski, J. Buchanan, C. Caillol, S. Dasu, L. Dodd, S. Duric, B. Gomber, M. Grothe, M. Herndon, A. Hervé, U. Hussain, P. Klabbers, A. Lanaro, A. Levine, K. Long, R. Loveless, T. Ruggles, A. Savin, N. Smith, W. H. Smith, D. Taylor, N. Woods, CMS Collaboration

**Affiliations:** 1 Yerevan Physics Institute, Yerevan, Armenia; 2 Institut für Hochenergiephysik, Wien, Austria; 3 Institute for Nuclear Problems, Minsk, Belarus; 4 Universiteit Antwerpen, Antwerpen, Belgium; 5 Vrije Universiteit Brussel, Brussel, Belgium; 6 Université Libre de Bruxelles, Bruxelles, Belgium; 7 Ghent University, Ghent, Belgium; 8 Université Catholique de Louvain, Louvain-la-Neuve, Belgium; 9 Centro Brasileiro de Pesquisas Fisicas, Rio de Janeiro, Brazil; 10 Universidade do Estado do Rio de Janeiro, Rio de Janeiro, Brazil; 11 Universidade Estadual Paulista, Universidade Federal do ABC, São Paulo, Brazil; 12 Institute for Nuclear Research and Nuclear Energy, Bulgarian Academy of Sciences, Sofia, Bulgaria; 13 University of Sofia, Sofia, Bulgaria; 14 Beihang University, Beijing, China; 15 Institute of High Energy Physics, Beijing, China; 16 State Key Laboratory of Nuclear Physics and Technology, Peking University, Beijing, China; 17 Tsinghua University, Beijing, China; 18 Universidad de Los Andes, Bogota, Colombia; 19 Faculty of Electrical Engineering, Mechanical Engineering and Naval Architecture, University of Split, Split, Croatia; 20 Faculty of Science, University of Split, Split, Croatia; 21 Institute Rudjer Boskovic, Zagreb, Croatia; 22 University of Cyprus, Nicosia, Cyprus; 23 Charles University, Prague, Czech Republic; 24 Universidad San Francisco de Quito, Quito, Ecuador; 25 Academy of Scientific Research and Technology of the Arab Republic of Egypt, Egyptian Network of High Energy Physics, Cairo, Egypt; 26 National Institute of Chemical Physics and Biophysics, Tallinn, Estonia; 27 Department of Physics, University of Helsinki, Helsinki, Finland; 28 Helsinki Institute of Physics, Helsinki, Finland; 29 Lappeenranta University of Technology, Lappeenranta, Finland; 30 IRFU, CEA, Université Paris-Saclay, Gif-sur-Yvette, France; 31 Laboratoire Leprince-Ringuet, Ecole polytechnique, CNRS/IN2P3, Université Paris-Saclay, Palaiseau, France; 32 Université de Strasbourg, CNRS, IPHC UMR 7178, 67000 Strasbourg, France; 33 Centre de Calcul de l’Institut National de Physique Nucleaire et de Physique des Particules, CNRS/IN2P3, Villeurbanne, France; 34 Université de Lyon, Université Claude Bernard Lyon 1, CNRS-IN2P3, Institut de Physique Nucléaire de Lyon, Villeurbanne, France; 35 Georgian Technical University, Tbilisi, Georgia; 36 Tbilisi State University, Tbilisi, Georgia; 37 RWTH Aachen University, I. Physikalisches Institut, Aachen, Germany; 38 RWTH Aachen University, III. Physikalisches Institut A, Aachen, Germany; 39 RWTH Aachen University, III. Physikalisches Institut B, Aachen, Germany; 40 Deutsches Elektronen-Synchrotron, Hamburg, Germany; 41 University of Hamburg, Hamburg, Germany; 42 Institut für Experimentelle Kernphysik, Karlsruhe, Germany; 43 Institute of Nuclear and Particle Physics (INPP), NCSR Demokritos, Aghia Paraskevi, Greece; 44 National and Kapodistrian University of Athens, Athens, Greece; 45 National Technical University of Athens, Athens, Greece; 46 University of Ioánnina, Ioánnina, Greece; 47 MTA-ELTE Lendület CMS Particle and Nuclear Physics Group, Eötvös Loránd University, Budapest, Hungary; 48 Wigner Research Centre for Physics, Budapest, Hungary; 49 Institute of Nuclear Research ATOMKI, Debrecen, Hungary; 50 Institute of Physics, University of Debrecen, Debrecen, Hungary; 51 Indian Institute of Science (IISc), Bangalore, India; 52 National Institute of Science Education and Research, Bhubaneswar, India; 53 Panjab University, Chandigarh, India; 54 University of Delhi, Delhi, India; 55 Saha Institute of Nuclear Physics, HBNI, Kolkata, India; 56 Indian Institute of Technology Madras, Madras, India; 57 Bhabha Atomic Research Centre, Mumbai, India; 58 Tata Institute of Fundamental Research-A, Mumbai, India; 59 Tata Institute of Fundamental Research-B, Mumbai, India; 60 Indian Institute of Science Education and Research (IISER), Pune, India; 61 Institute for Research in Fundamental Sciences (IPM), Tehran, Iran; 62 University College Dublin, Dublin, Ireland; 63 INFN Sezione di Bari, Università di Bari, Politecnico di Bari, Bari, Italy; 64 INFN Sezione di Bologna, Università di Bologna, Bologna, Italy; 65 INFN Sezione di Catania, Università di Catania, Catania, Italy; 66 INFN Sezione di Firenze, Università di Firenze, Firenze, Italy; 67 INFN Laboratori Nazionali di Frascati, Frascati, Italy; 68 INFN Sezione di Genova, Università di Genova, Genoa, Italy; 69 INFN Sezione di Milano-Bicocca, Università di Milano-Bicocca, Milan, Italy; 70 INFN Sezione di Napoli, Università di Napoli ’Federico II’ , Napoli, Italy, Università della Basilicata, Potenza, Italy, Università G. Marconi, Rome, Italy; 71 INFN Sezione di Padova, Università di Padova, Padova, Italy, Università di Trento, Trento, Italy; 72 INFN Sezione di Pavia, Università di Pavia, Pavia, Italy; 73 INFN Sezione di Perugia, Università di Perugia, Perugia, Italy; 74 INFN Sezione di Pisa, Università di Pisa, Scuola Normale Superiore di Pisa, Pisa, Italy; 75 INFN Sezione di Roma, Sapienza Università di Roma, Rome, Italy; 76 INFN Sezione di Torino, Università di Torino, Turin, Italy, Università del Piemonte Orientale, Novara, Italy; 77 INFN Sezione di Trieste, Università di Trieste, Trieste, Italy; 78 Kyungpook National University, Daegu, Korea; 79 Chonbuk National University, Jeonju, Korea; 80 Chonnam National University, Institute for Universe and Elementary Particles, Kwangju, Korea; 81 Hanyang University, Seoul, Korea; 82 Korea University, Seoul, Korea; 83 Seoul National University, Seoul, Korea; 84 University of Seoul, Seoul, Korea; 85 Sungkyunkwan University, Suwon, Korea; 86 Vilnius University, Vilnius, Lithuania; 87 National Centre for Particle Physics, Universiti Malaya, Kuala Lumpur, Malaysia; 88 Centro de Investigacion y de Estudios Avanzados del IPN, Mexico City, Mexico; 89 Universidad Iberoamericana, Mexico City, Mexico; 90 Benemerita Universidad Autonoma de Puebla, Puebla, Mexico; 91 Universidad Autónoma de San Luis Potosí, San Luis Potosí, Mexico; 92 University of Auckland, Auckland, New Zealand; 93 University of Canterbury, Christchurch, New Zealand; 94 National Centre for Physics, Quaid-I-Azam University, Islamabad, Pakistan; 95 National Centre for Nuclear Research, Swierk, Poland; 96 Institute of Experimental Physics, Faculty of Physics, University of Warsaw, Warsaw, Poland; 97 Laboratório de Instrumentação e Física Experimental de Partículas, Lisboa, Portugal; 98 Joint Institute for Nuclear Research, Dubna, Russia; 99 Petersburg Nuclear Physics Institute, Gatchina, (St. Petersburg), Russia; 100 Institute for Nuclear Research, Moscow, Russia; 101 Institute for Theoretical and Experimental Physics, Moscow, Russia; 102 Moscow Institute of Physics and Technology, Moscow, Russia; 103 National Research Nuclear University ‘Moscow Engineering Physics Institute’ (MEPhI), Moscow, Russia; 104 P.N. Lebedev Physical Institute, Moscow, Russia; 105 Skobeltsyn Institute of Nuclear Physics, Lomonosov Moscow State University, Moscow, Russia; 106 Novosibirsk State University (NSU), Novosibirsk, Russia; 107 State Research Center of Russian Federation, Institute for High Energy Physics of NRC&quot;Kurchatov Institute&quot;, Protvino, Russia; 108 University of Belgrade, Faculty of Physics and Vinca Institute of Nuclear Sciences, Belgrade, Serbia; 109 Centro de Investigaciones Energéticas Medioambientales y Tecnológicas (CIEMAT), Madrid, Spain; 110 Universidad Autónoma de Madrid, Madrid, Spain; 111 Universidad de Oviedo, Oviedo, Spain; 112 Instituto de Física de Cantabria (IFCA), CSIC-Universidad de Cantabria, Santander, Spain; 113 CERN, European Organization for Nuclear Research, Geneva, Switzerland; 114 Paul Scherrer Institut, Villigen, Switzerland; 115 ETH Zurich, Institute for Particle Physics and Astrophysics (IPA), Zurich, Switzerland; 116 Universität Zürich, Zurich, Switzerland; 117 National Central University, Chung-Li, Taiwan; 118 National Taiwan University (NTU), Taipei, Taiwan; 119 Department of Physics, Faculty of Science, Chulalongkorn University, Bangkok, Thailand; 120 Physics Department, Science and Art Faculty, Çukurova University, Adana, Turkey; 121 Physics Department, Middle East Technical University, Ankara, Turkey; 122 Bogazici University, Istanbul, Turkey; 123 Istanbul Technical University, Istanbul, Turkey; 124 Institute for Scintillation Materials, National Academy of Science of Ukraine, Kharkov, Ukraine; 125 National Scientific Center, Kharkov Institute of Physics and Technology, Kharkov, Ukraine; 126 University of Bristol, Bristol, UK; 127 Rutherford Appleton Laboratory, Didcot, UK; 128 Imperial College, London, UK; 129 Brunel University, Uxbridge, UK; 130 Baylor University, Waco, USA; 131 Catholic University of America, Washington, DC USA; 132 The University of Alabama, Tuscaloosa, USA; 133 Boston University, Boston, USA; 134 Brown University, Providence, USA; 135 University of California, Davis, Davis, USA; 136 University of California, Los Angeles, USA; 137 University of California, Riverside, Riverside, USA; 138 University of California, San Diego, La Jolla, USA; 139 Department of Physics, University of California, Santa Barbara, Santa Barbara, USA; 140 California Institute of Technology, Pasadena, USA; 141 Carnegie Mellon University, Pittsburgh, USA; 142 University of Colorado Boulder, Boulder, USA; 143 Cornell University, Ithaca, USA; 144 Fermi National Accelerator Laboratory, Batavia, USA; 145 University of Florida, Gainesville, USA; 146 Florida International University, Miami, USA; 147 Florida State University, Tallahassee, USA; 148 Florida Institute of Technology, Melbourne, USA; 149 University of Illinois at Chicago (UIC), Chicago, USA; 150 The University of Iowa, Iowa City, USA; 151 Johns Hopkins University, Baltimore, USA; 152 The University of Kansas, Lawrence, USA; 153 Kansas State University, Manhattan, USA; 154 Lawrence Livermore National Laboratory, Livermore, USA; 155 University of Maryland, College Park, USA; 156 Massachusetts Institute of Technology, Cambridge, USA; 157 University of Minnesota, Minneapolis, USA; 158 University of Mississippi, Oxford, USA; 159 University of Nebraska-Lincoln, Lincoln, USA; 160 State University of New York at Buffalo, Buffalo, USA; 161 Northeastern University, Boston, USA; 162 Northwestern University, Evanston, USA; 163 University of Notre Dame, Notre Dame, USA; 164 The Ohio State University, Columbus, USA; 165 Princeton University, Princeton, USA; 166 University of Puerto Rico, Mayaguez, USA; 167 Purdue University, West Lafayette, USA; 168 Purdue University Northwest, Hammond, USA; 169 Rice University, Houston, USA; 170 University of Rochester, Rochester, USA; 171 The Rockefeller University, New York, USA; 172 Rutgers, The State University of New Jersey, Piscataway, USA; 173 University of Tennessee, Knoxville, USA; 174 Texas A & M University, College Station, USA; 175 Texas Tech University, Lubbock, USA; 176 Vanderbilt University, Nashville, USA; 177 University of Virginia, Charlottesville, USA; 178 Wayne State University, Detroit, USA; 179 University of Wisconsin-Madison, Madison, WI USA; 180 CERN, 1211 Geneva 23, Switzerland

## Abstract

The nuclear modification factors of ${\mathrm {J}/\psi }$ and $\psi \text {(2S)}$ mesons are measured in $\text {PbPb}$ collisions at a centre-of-mass energy per nucleon pair of $\sqrt{\smash [b]{s_{_{\text {NN}}}}} = 5.02\,\text {Te}\text {V}$. The analysis is based on $\text {PbPb}$ and $\mathrm {p}\mathrm {p}$ data samples collected by CMS at the LHC in 2015, corresponding to integrated luminosities of 464$\,\mu \mathrm {b}^{-1}$ and 28$\,\text {pb}^\text {-1}$, respectively. The measurements are performed in the dimuon rapidity range of $|y | < 2.4$ as a function of centrality, rapidity, and transverse momentum ($p_{\mathrm {T}}$ ) from $p_{\mathrm {T}} =3$ ${\,\text {Ge}\text {V}/}\text {c}$ in the most forward region and up to 50${\,\text {Ge}\text {V}/}\text {c}$. Both prompt and nonprompt (coming from b hadron decays) ${\mathrm {J}/\psi }$ mesons are observed to be increasingly suppressed with centrality, with a magnitude similar to the one observed at $\sqrt{\smash [b]{s_{_{\text {NN}}}}} = 2.76\,\text {Te}\text {V}$ for the two ${\mathrm {J}/\psi }$ meson components. No dependence on rapidity is observed for either prompt or nonprompt ${\mathrm {J}/\psi }$ mesons. An indication of a lower prompt ${\mathrm {J}/\psi }$ meson suppression at $p_{\mathrm {T}} > 25$ ${\,\text {Ge}\text {V}/}\text {c}$ is seen with respect to that observed at intermediate $p_{\mathrm {T}}$. The prompt $\psi \text {(2S)}$ meson yield is found to be more suppressed than that of the prompt ${\mathrm {J}/\psi }$ mesons in the entire $p_{\mathrm {T}}$ range.

## Introduction

Quarkonium production in heavy ion collisions has a rich history. In their original article [1], Matsui and Satz proposed that Debye color screening of the heavy-quark potential in a hot medium prevents the production of ${\mathrm {J}/\psi }$ mesons (and this applies also to other heavy-quark bound states such as $\psi \text {(2S)}$, and $\mathrm {\varUpsilon (1S)}$ mesons [2]). Consequently, the suppression of quarkonium yields in heavy ion collisions, relative to those in $\mathrm {p}\mathrm {p}$ collisions, has long been considered to be a sensitive probe of deconfinement and quark-gluon plasma formation. The ${\mathrm {J}/\psi }$ meson suppression observed in $\text {PbPb}$ collisions at the CERN SPS [3] and $\text {AuAu}$ collisions at the BNL RHIC [4] is compatible with this picture. Similarly, the disappearance of $\mathrm {\varUpsilon }$ resonances in $\text {PbPb}$ collisions at the CERN LHC [5, 6] is consistent with the Debye screening scenario.

When produced abundantly in a single heavy ion collision, uncorrelated heavy quarks may combine to form quarkonia states in the medium [7, 8]. This additional source of quarkonium, commonly referred to as *recombination*, would enhance its production in heavy ion collisions, in contradistinction with the Debye screening scenario. Signs of this effect can be seen in the recent results from the ALICE Collaboration at the LHC [9, 10], which measured a weaker ${\mathrm {J}/\psi }$ meson suppression than at RHIC [4, 11], despite the higher medium energy density. Note that recombination is only expected to affect charmonium production at low transverse momenta ($p_\text {T}$), typically for values smaller than the charmonium mass ($p_{\mathrm {T}} \lesssim m_{\psi } \, c$), where the number of charm quarks initially produced in the collision is the largest [8].

At large $p_{\mathrm {T}}$, other mechanisms may contribute to charmonium suppression. Until recently, no quarkonium results were available at high $p_{\mathrm {T}}$, because of kinematic constraints at the SPS and too low counting rates at RHIC. At the LHC, a strong ${\mathrm {J}/\psi }$ suppression has been measured up to $p_{\mathrm {T}} = 30$ ${\,\text {Ge}\text {V}/}\text {c}$ by the CMS Collaboration [12] in $\text {PbPb}$ collisions at a centre-of-mass energy per nucleon pair of $\sqrt{\smash [b]{s_{_{\text {NN}}}}} = 2.76\,\text {Te}\text {V}$. Results at $5.02\,\text {Te}\text {V}$ have also been reported, up to $p_{\mathrm {T}} = 10$ ${\,\text {Ge}\text {V}/}\text {c}$, by the ALICE Collaboration [10]. According to Refs. [13, 14], quarkonium suppression by Debye screening may occur even at high $p_{\mathrm {T}}$. At the same time, when $p_{\mathrm {T}} \gg m_{\psi } \, c$, heavy quarkonium is likely to be produced by parton fragmentation, hence it should rather be sensitive to the parton energy loss in the quark-gluon plasma. The similarity of ${\mathrm {J}/\psi }$ meson suppression with the quenching of jets, light hadrons, and D mesons supports this picture [12, 15, 16].

At the LHC, the inclusive ${\mathrm {J}/\psi }$ meson yield also contains a significant *nonprompt* contribution coming from $\mathrm {b}$ hadron decays [17–19]. The nonprompt ${\mathrm {J}/\psi }$ component should reflect medium effects on $\mathrm {b}$ hadron production in heavy ion collisions, such as b quark energy loss. Measuring both prompt and nonprompt ${\mathrm {J}/\psi }$ meson production in $\text {PbPb}$ collisions thus offers the opportunity to study both hidden charm and open beauty production in the same data sample.

In this paper we report on a new measurement of the prompt and nonprompt ${\mathrm {J}/\psi }$ and $\psi \text {(2S)}$ nuclear modification factors ($R_{\text {AA}}$) using $\text {PbPb}$ data, collected at the end of 2015 with the CMS experiment at $\sqrt{\smash [b]{s_{_{\text {NN}}}}} = 5.02\,\text {Te}\text {V}$. The analysis is performed via the dimuon decay channel. The results are compared to those obtained at 2.76$\,\text {Te}\text {V}$  [12]. The larger integrated luminosities allow for more precise and more differential measurements of $R_{\text {AA}}$, as functions of centrality, rapidity (*y*), and $p_{\mathrm {T}}$ up to 50${\,\text {Ge}\text {V}/}\text {c}$.

## The CMS detector

The central feature of the CMS apparatus is a superconducting solenoid of 6$\text {\,m}$ internal diameter, providing a magnetic field of 3.8$\text {\,T}$. Within the solenoid volume are a silicon pixel and strip tracker, a lead tungstate crystal electromagnetic calorimeter, and a brass and scintillator hadron calorimeter, each composed of a barrel and two endcap sections. Forward calorimeters extend the coverage provided by the barrel and endcap detectors. Muons are measured in the pseudorapidity range $|\eta | < 2.4$ in gas-ionisation detectors embedded in the steel flux-return yoke outside the solenoid, with detection planes made using three technologies: drift tubes, cathode strip chambers, and resistive-plate chambers. The hadron forward (HF) calorimeters use steel as an absorber and quartz fibres as the sensitive material. The two HF calorimeters are located 11.2$\text {\,m}$ from the interaction region, one on each side, and together they provide coverage in the range $2.9< |\eta | < 5.2$. They also serve as luminosity monitors. Two beam pick-up timing detectors are located at 175$\text {\,m}$ on both sides of the interaction point, and provide information about the timing structure of the LHC beam. Events of interest are selected using a two-tiered trigger system [20]. The first level (L1), composed of custom hardware processors, uses information from the calorimeters and muon detectors to select events. The second level, known as the high-level trigger (HLT), consists of a farm of processors running a version of the full event reconstruction software optimised for fast processing. A more detailed description of the CMS detector, together with a definition of the coordinate system used and the relevant kinematic variables, can be found in Ref. [21].

For $\mathrm {p}\mathrm {p}$ data the vertices are reconstructed with a deterministic annealing vertex fitting algorithm using all of the fully reconstructed tracks [22]. The physics objects used to determine the primary vertex are defined based on a jet finding algorithm [23, 24] applied to all charged tracks associated with the vertex, plus the corresponding associated missing transverse momentum. The reconstructed vertex with the largest value of summed physics object $p_{\mathrm {T}} ^2$ is taken to be the primary $\mathrm {p}\mathrm {p}$ interaction vertex. In the case of $\text {PbPb}$ data, a single primary vertex is reconstructed using a gap clustering algorithm [22], using pixel tracks only.

## Data selection

### Event selection

Hadronic collisions are selected offline using information from the HF calorimeters. In order to select $\text {PbPb}$ collisions, at least three towers with energy deposits above 3 GeV are required in each of the HF calorimeters, both at forward and backward rapidities. A primary vertex reconstructed with at least two tracks is also required. In addition, a filter on the compatibility of the silicon pixel cluster width and the vertex position is applied [25]. The combined efficiency for this event selection, including the remaining non-hadronic contamination, is $(99 \pm 2)$%. Values higher than 100% are possible, reflecting the possible presence of ultra-peripheral (i.e. non-hadronic) collisions in the selected event sample.

The $\text {PbPb}$ sample is divided into bins of collision centrality, which is a measure of the degree of overlap of the colliding nuclei and is related to the number of participating nucleons ($N_{\text {part}}$). Centrality is defined as the percentile of the inelastic hadronic cross section corresponding to a HF energy deposit above a certain threshold [26]. The most central (highest HF energy deposit) and most peripheral (lowest HF energy deposit) centrality bins used in the analysis are 0–5% and 70–100% respectively. Variables related to the centrality, such as $N_{\text {part}}$ and the nuclear overlap function ($T_{\text {AA}}$) [27], are estimated using a Glauber model simulation described in Ref. [28].

The $\mathrm {p}\mathrm {p}$ and $\text {PbPb}$ data sets correspond to integrated luminosities of 28.0$\,\text {pb}^\text {-1}$ and 464$\,\mu \mathrm {b}^{-1}$, respectively. Both ${\mathrm {J}/\psi }$ and $\psi \text {(2S)}$ mesons are reconstructed using their dimuon decay channel. The dimuon events were selected online by the L1 trigger system, requiring two tracks in the muon detectors with no explicit momentum threshold, in coincidence with a bunch crossing identified by beam pick-up timing detectors. No additional selection was applied by the HLT. Because of the high rate of the most central dimuon events, a prescale was applied at the HLT level during part of the PbPb data taking: as a consequence only 79% of all the dimuon events were recorded, resulting in an effective luminosity of 368$\,\mu \mathrm {b}^{-1}$. For peripheral events we were able to sample the entire integrated luminosity of 464$\,\mu \mathrm {b}^{-1}$. This was done by adding an additional requirement that events be in the centrality range of 30–100% to the dimuon trigger. The prescaled data sample is used for the results integrated over centrality and those in the centrality range 0–30%, while for the results in the 30–100% range the data sample with 464$\,\mu \mathrm {b}^{-1}$ was used instead. The results reported in this paper are unaffected by the small number of extra collisions potentially present in the collected events: the mean of the Poisson distribution of the number of collisions per bunch crossing (pileup), averaged over the full data sample, is approximately 0.9 for the $\mathrm {p}\mathrm {p}$ data and less than 0.01 for $\text {PbPb}$ collisions.

Simulated events are used to tune the muon selection criteria and the signal fitting parameters, as well as for acceptance and efficiency studies. These samples, produced using pythia 8.212 [29], and decaying the $\mathrm {b}$ hadrons with evtgen 1.3.0 [30], are embedded in a realistic $\text {PbPb}$ background event generated with hydjet 1.9 [31] and propagated through the CMS detector with Geant4  [32]. The prompt ${\mathrm {J}/\psi }$ is simulated unpolarised, a scenario in good agreement with pp measurements [33–35]. For nonprompt ${\mathrm {J}/\psi }$, the polarisation is the one predicted by evtgen, roughly $\lambda _{\theta } = 0.4$. The resulting events are processed through the trigger emulation and the event reconstruction sequences. The assumptions made on the quarkonium polarisation affect the computation of the acceptance. Quantitative estimates of the possible effect evaluated for several polarisation scenarios can be found in Refs. [36, 37]. While there are no measurements on quarkonium polarisations in $\text {PbPb}$ collisions, a study in $\mathrm {p}\mathrm {p}$ collisions as a function of the event activity [38] has not revealed significant changes. Therefore the effects of the ${\mathrm {J}/\psi }$ polarisation on the acceptance are not considered as systematic uncertainties.

### Muon selection

The muon reconstruction algorithm starts by finding tracks in the muon detectors, which are then fitted together with tracks reconstructed in the silicon tracker. Kinematic selections are imposed to single muons so that their combined trigger, reconstruction and identification efficiency stays above 10%. These selections are: $p_{\mathrm {T}} ^{\mu }>3.50{\,\text {Ge}\text {V}/}\text {c}$ for $|\eta ^{\mu } |<1.2$ and $p_{\mathrm {T}} ^{\mu }>1.89{\,\text {Ge}\text {V}/}\text {c}$ for $2.1<|\eta ^{\mu } |<2.4$, linearly interpolated in the intermediate $|\eta ^{\mu } |$ region. The muons are required to match the ones selected by the dimuon trigger, and *soft* muon selection criteria are applied to *global muons* (i.e. muons reconstructed using the combined information of the tracker and muon detectors), as defined in Ref. [39]. Matching muons to tracks measured in the silicon tracker results in a relative $p_{\mathrm {T}}$ resolution for muons between 1 and 2% for a typical muon in this analysis [39]. In order to remove cosmic and in-flight decay muons, the transverse and longitudinal distances of approach to the measured vertex of the muons entering in the analysis are required to be less than 0.3 and 20 cm, respectively. The probability that the two muon tracks originate from a common vertex is required to be larger than 1%, lowering the background from $\mathrm {b}$ and $\mathrm {c}$ hadron semileptonic decays.

## Signal extraction

Because of the long lifetime of $$\mathrm {b}$$ hadrons compared to that of $${\mathrm {J}/\psi }$$ mesons, the separation of the prompt and nonprompt $${\mathrm {J}/\psi }$$ components relies on the measurement of a secondary $$\mu ^+ \mu ^- $$ vertex displaced from the primary collision vertex. The $${\mathrm {J}/\psi }$$ mesons originating from the decay of $$\mathrm {b}$$ hadrons can be resolved using the pseudo-proper decay length [40] $$\ell _{{\mathrm {J}/\psi }} = L_{xyz} \, m_{{\mathrm {J}/\psi }} \, c / |p_{\mu \mu }|$$, where $$L_{xyz} $$ is the distance between the primary and dimuon vertices, $$m_{{\mathrm {J}/\psi }}$$ is the Particle Data Group [41] world average value of the $${\mathrm {J}/\psi }$$ meson mass (assumed for all dimuon candidates), and $$p_{\mu \mu }$$ is the dimuon momentum. Note that due to resolution effects and background dimuons the pseudo-proper decay length can take negative values. To measure the fraction of $${\mathrm {J}/\psi }$$ mesons coming from $$\mathrm {b}$$ hadron decays (the so-called nonprompt fraction), the invariant mass spectrum of $$ \mu ^+ \mu ^- $$ pairs and their $$\ell _{{\mathrm {J}/\psi }}$$ distribution are fitted using a two-dimensional (2D) extended unbinned maximum-likelihood fit. In order to obtain the parameters of the different components of the 2D probability density function (PDF), the invariant mass and the $$\ell _{{\mathrm {J}/\psi }}$$ distributions are fitted sequentially prior to the final 2D fits, as explained below. These fits are performed for each $$p_{\mathrm {T}}$$, rapidity and centrality bin of the analysis, and separately in $$\mathrm {p}\mathrm {p}$$ and $$\text {PbPb}$$ collisions.

The sum of two Crystal Ball functions [42], with different widths but common mean and tail parameters, is used to extract the nominal yield values from the $\mathrm {p}\mathrm {p}$ and $\text {PbPb}$ invariant mass distributions. The tail parameters, as well as the ratio of widths in the $\text {PbPb}$ case, are fixed to the values obtained from simulation. The background is described by a polynomial function of order *N*, where *N* is the lowest value that provides a good description of the data, and is determined by performing a log-likelihood ratio test between polynomials of different orders, in each analysis bin, while keeping the tail and width ratio parameters fixed. The order of the polynomial is chosen in such a way that increasing the order does not significantly improve the quality of the fit. The typical order of the polynomial is 1 for most of the analysis bins. The invariant mass signal and background parameters are obtained in an initial fit of the invariant mass distribution only and then fixed on the 2D fits of mass and $\ell _{{\mathrm {J}/\psi }}$ distributions, while the number of extracted ${\mathrm {J}/\psi }$ mesons and background dimuons are left as free parameters.

**Fig. 1:**
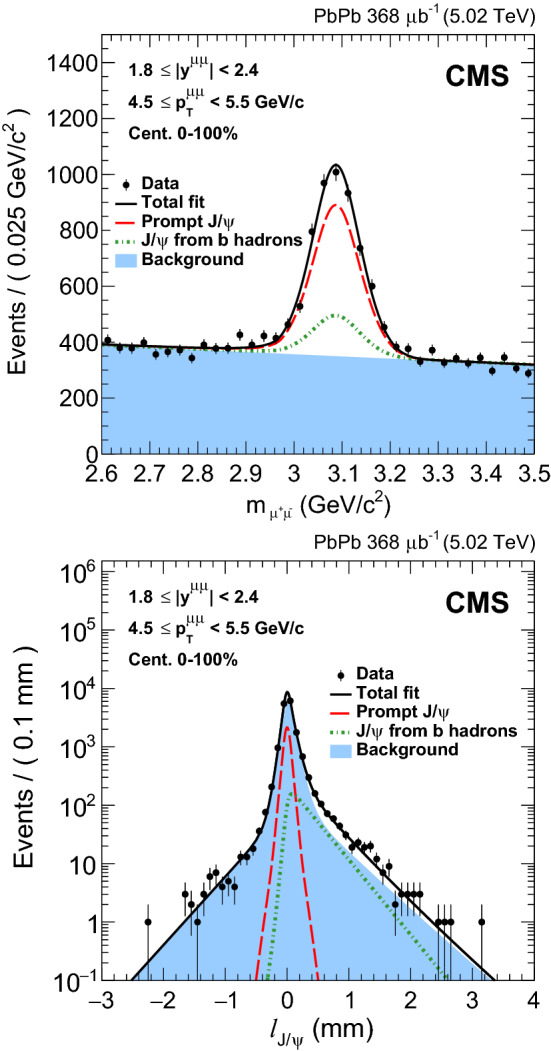
Invariant mass spectrum of $\mu ^+ \mu ^-$ pairs (upper) and pseudo-proper decay length distribution (lower) in $\text {PbPb}$ collisions for $1.8<|y |<2.4$, $4.5<p_{\mathrm {T}} <5.5{\,\text {Ge}\text {V}/}\text {c}$, for all centralities. The result of the fit described in the text is also shown

The prompt, nonprompt, and background components of the $\ell _{{\mathrm {J}/\psi }}$ distributions are parameterised using collision data and Monte Carlo (MC) simulated events, and the signal and background contributions unfolded with the ${}_{s}\mathcal {P}lot$ technique [43]. In the context of this analysis, this technique uses the invariant mass signal and background PDFs to discriminate signal from background in the $\ell _{{\mathrm {J}/\psi }}$ distribution. The $\ell _{{\mathrm {J}/\psi }}$ per-event uncertainty distributions of signal and background, provided by the reconstruction algorithm of primary and secondary vertices, are extracted from data and used as templates. The $\ell _{{\mathrm {J}/\psi }}$ resolution is also obtained from the data by fitting the distribution of events with $\ell _{{\mathrm {J}/\psi }} < 0$ with a combination of three Gaussian functions. The resolution varies event-by-event, so the per-event uncertainty is used as the width of the Gaussian function that describes the core. To take into account the difference on the per-event uncertainty distributions of signal and background dimuons, the resolution PDF is multiplied by the per-event uncertainty distribution of signal and background dimuons separately. All the resolution parameters are fixed in the 2D fits. The $\mathrm {b}$ hadron decay length is allowed to float freely in the fit, and it is initialised to the value extracted by fitting the $\ell _{{\mathrm {J}/\psi }}$ distribution of nonprompt ${\mathrm {J}/\psi }$ mesons from a MC sample with an exponential decay function, at generator level. The $\ell _{{\mathrm {J}/\psi }}$ distribution of background dimuons is obtained from fits to the data, using an empirical combination of exponential functions. The parameters of the $\ell _{{\mathrm {J}/\psi }}$ background distribution are also fixed in the 2D fits. Finally, the number of extracted ${\mathrm {J}/\psi }$ mesons, the number of background dimuons and the nonprompt fraction are extracted from the 2D fits. An example of a 2D fit of the invariant mass and pseudo-proper decay length for the $\text {PbPb}$ data is shown in Fig. 1 for a representative analysis bin.

## Acceptance and efficiency corrections

Correction factors are applied to all results to account for detector acceptance, trigger, reconstruction, and selection efficiencies of the $\mu ^+ \mu ^-$ pairs. The corrections are derived from prompt and nonprompt ${\mathrm {J}/\psi }$ meson MC samples in $\mathrm {p}\mathrm {p}$ and $\text {PbPb}$, and are evaluated in the same bins of $p_{\mathrm {T}}$, centrality, and rapidity used in the $R_{\text {AA}}$ and cross section analyses. The prompt and nonprompt ${\mathrm {J}/\psi }$ meson $p_{\mathrm {T}}$ distributions in bins of rapidity in MC samples are compared to those in data, and the ratios of data over MC are used to weight the MC ${\mathrm {J}/\psi }$ distributions to describe the data better. This weighting accounts for possible mis-modelling of ${\mathrm {J}/\psi }$ kinematics in MC. The acceptance in a given analysis bin is defined as the fraction of generated ${\mathrm {J}/\psi }$ mesons in that bin which decay into two muons entering the kinematic limits defined above, and reflects the geometrical coverage of the CMS detector. The value of the acceptance correction ranges from 4 to 70%, depending on the dimuon $p_{\mathrm {T}}$, both for prompt and nonprompt ${\mathrm {J}/\psi }$ mesons in $\mathrm {p}\mathrm {p}$ and $\text {PbPb}$ collisions. The efficiency in a given analysis bin is defined as the ratio of the number of reconstructed ${\mathrm {J}/\psi }$ mesons in which both muons pass the analysis selection and the number of generated ${\mathrm {J}/\psi }$ mesons in which both muons pass the analysis selection. The efficiency correction depends on the dimuon $p_{\mathrm {T}}$, rapidity and event centrality, and ranges from 20 to 75% (15 to 75%) for prompt (nonprompt) ${\mathrm {J}/\psi }$ mesons in $\text {PbPb}$ data, and from 40 to 85% for both prompt and nonprompt ${\mathrm {J}/\psi }$ mesons in $\mathrm {p}\mathrm {p}$ data. The efficiency is lower at low than at high $p_{\mathrm {T}}$, and it decreases from mid to forward rapidity; it is also lower for central than peripheral events. The individual components of the efficiency (tracking reconstruction, standalone muon reconstruction, global muon fit, muon identification and selection, and triggering) are also measured using single muons from ${\mathrm {J}/\psi }$ meson decays in both simulated and collision data, using the *tag-and-probe* (T&P) technique [36, 44]. The values obtained from data and simulation are seen to differ only for the muon trigger efficiency and the ratio of the data over simulated efficiencies is used as a correction factor for the efficiency. The correction factor for dimuons is at most 1.35 (1.38) for the $\mathrm {p}\mathrm {p}$ ($\text {PbPb}$) efficiency in the $3< p_{\mathrm {T}} < 4.5$ ${\,\text {Ge}\text {V}/}\text {c}$ and forward rapidity bin, but the $p_{\mathrm {T}}$ and rapidity integrated value of the correction is about 1.03. The other T&P efficiency components are compatible, hence only used as a cross-check, as well as to estimate systematic uncertainties.

## Systematic uncertainties

The systematic uncertainties in these measurements arise from the invariant mass signal and background fitting model assumptions, the parameterisation of the $\ell _{{\mathrm {J}/\psi }}$ distribution, the acceptance and efficiency computation, and sample normalisation (integrated luminosity in $\mathrm {p}\mathrm {p}$ data, counting of the equivalent number of minimum bias events in $\text {PbPb}$, and nuclear overlap function). These systematic uncertainties are derived separately for $\mathrm {p}\mathrm {p}$ and $\text {PbPb}$ results, and the total systematic uncertainty is computed as the quadratic sum of the partial terms.

The systematic uncertainty due to each component of the 2D fits is estimated from the difference between the nominal value and the result obtained with the variations of the different components mentioned below, in the extracted number of prompt and nonprompt ${\mathrm {J}/\psi }$ mesons, or nonprompt fraction separately. In the following, the typical uncertainty is given for the observable on which each source has the biggest impact.

In order to determine the uncertainty associated with the invariant mass fitting procedure, the signal and background PDFs are independently varied, in each analysis bin. For the uncertainty in the signal, the parameters that were fixed in the nominal fits are left free with a certain constraint. The constraint for each parameter is determined from fits to the data, by leaving only one of the parameters free, and it is chosen as the root mean square of the variations over the different analysis bins. A different signal shape is also used: a Crystal Ball function plus a Gaussian function, with the CB tail parameters, as well as the ratio of widths in the PbPb case, again fixed from MC. The dominant uncertainty comes from the variation of the signal shape, yielding values for the number of extracted nonprompt ${\mathrm {J}/\psi }$ mesons ranging from 0.1 to 2.9% (0.3–5.5%) in $\mathrm {p}\mathrm {p}$ ($\text {PbPb}$) data. For the background model, the following changes are considered, while keeping the nominal signal shape. First, the log-likelihood ratio tests are done again with two variations of the threshold used to choose the order of the polynomial function in each analysis bin. Also the fitted mass range is varied. Finally, an exponential of a polynomial function is also used. The dominant uncertainty in the background model arises from the assumed shape (invariant mass range) in $\mathrm {p}\mathrm {p}$ ($\text {PbPb}$) data. The corresponding uncertainty ranges from 0.1 to 2.1% (0.1–2.8%). The maximum difference of each of these variations, in each analysis bin and separately for the signal and the background, is taken as an independent systematic uncertainty.

**Fig. 2:**
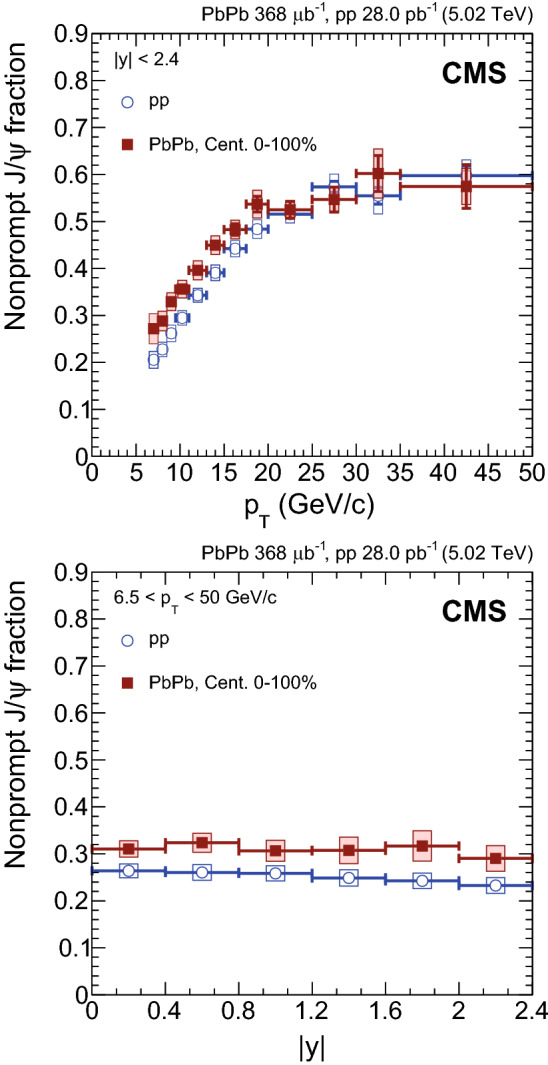
Fraction of ${\mathrm {J}/\psi }$ mesons coming from the decay of b hadrons, i.e. nonprompt ${\mathrm {J}/\psi }$ meson fraction, as a function of dimuon $p_{\mathrm {T}}$ (upper) and rapidity (lower) for $\mathrm {p}\mathrm {p}$ and $\text {PbPb}$ collisions, for all centralities. The bars (boxes) represent statistical (systematic) point-by-point uncertainties

**Fig. 3:**
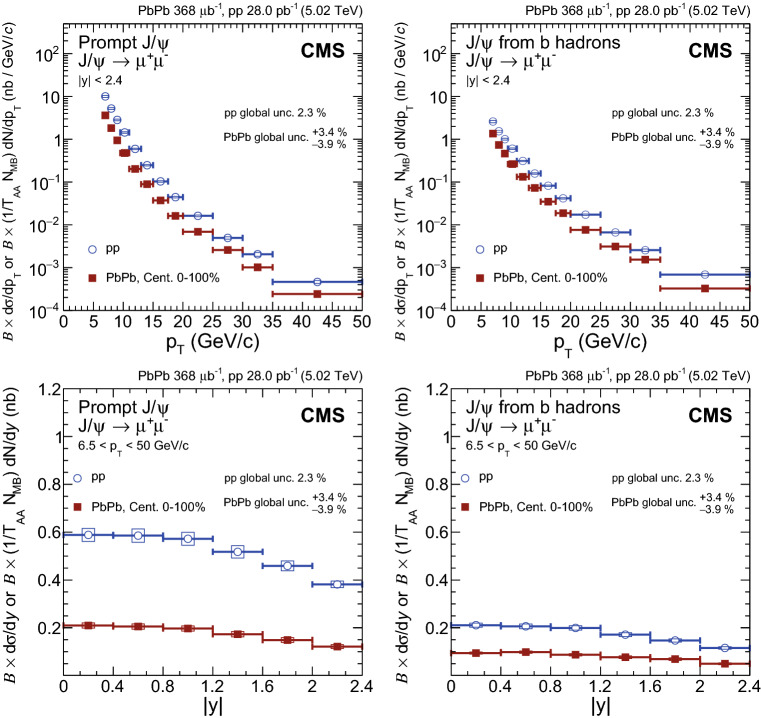
Differential cross section of prompt ${\mathrm {J}/\psi }$ mesons (left) and ${\mathrm {J}/\psi }$ mesons from b hadrons (nonprompt ${\mathrm {J}/\psi }$) (right) decaying into two muons as a function of dimuon $p_{\mathrm {T}}$ (upper) and rapidity (lower) in $\mathrm {p}\mathrm {p}$ and $\text {PbPb}$ collisions. The $\text {PbPb}$ cross sections are normalised by $T_{\text {AA}}$ for direct comparison. The bars (boxes) represent statistical (systematic) point-by-point uncertainties, while global uncertainties are written on the plots

**Fig. 4:**
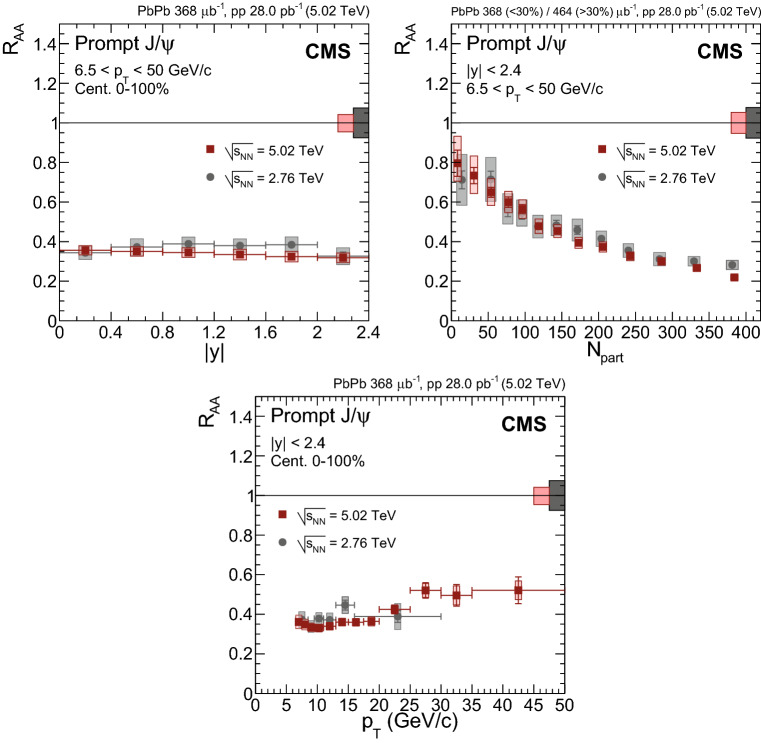
Nuclear modification factor of prompt ${\mathrm {J}/\psi }$ mesons as a function of dimuon rapidity (upper left), $N_{\text {part}}$ (upper right) and dimuon $p_{\mathrm {T}}$ (lower) at $\sqrt{\smash [b]{s_{_{\text {NN}}}}} = 5.02$ $\,\text {Te}\text {V}$. For the results as a function of $N_{\text {part}}$ the most central bin corresponds to 0–5%, and the most peripheral one to 70–100%. Results obtained at 2.76$\,\text {Te}\text {V}$ are overlaid for comparison [12]. The bars (boxes) represent statistical (systematic) point-by-point uncertainties. The boxes plotted at $R_{\text {AA}} =1$ indicate the size of the global relative uncertainties

For the $\ell _{{\mathrm {J}/\psi }}$ distribution fitting procedure, four independent variations of the different components entering in the 2D fits are considered. For the $\ell _{{\mathrm {J}/\psi }}$ uncertainty distribution, instead of using the distributions corresponding to signal and background, the total distribution is assumed. The contribution to the systematic uncertainty in the number of extracted nonprompt ${\mathrm {J}/\psi }$ mesons ranges from 0.3 to 2% (0.3–9.5%) in $\mathrm {p}\mathrm {p}$ ($\text {PbPb}$) data. The $\ell _{{\mathrm {J}/\psi }}$ resolution obtained from prompt ${\mathrm {J}/\psi }$ meson MC is used instead of that evaluated from data. The corresponding uncertainty in the nonprompt fraction ranges from 1 to 5% (1–11%) in $\mathrm {p}\mathrm {p}$ ($\text {PbPb}$) data. A nonprompt ${\mathrm {J}/\psi }$ meson MC template replaces the exponential decay function for the $\mathrm {b}$ hadron decay length. In this case, the contribution of this source to the systematic uncertainty in the nonprompt ${\mathrm {J}/\psi }$ yield ranges from 0.2 to 8% (0.2–20%) in $\mathrm {p}\mathrm {p}$ ($\text {PbPb}$) data. A template of the $\ell _{{\mathrm {J}/\psi }}$ distribution of background dimuons obtained from the data is used to describe the background, instead of the empirical combination of exponential functions. This variation has an impact on the nonprompt ${\mathrm {J}/\psi }$ yield ranging from 0.1 to 1.3% (0.2–22%) in $\mathrm {p}\mathrm {p}$ ($\text {PbPb}$) data. Therefore the dominant sources of uncertainty in the $\ell _{{\mathrm {J}/\psi }}$ fitting are the background parameterisation and the MC template for the nonprompt signal. They have an important impact on the nonprompt ${\mathrm {J}/\psi }$ meson yield, especially at the lowest $p_{\mathrm {T}}$ reached in this analysis for the most central events in $\text {PbPb}$ collisions. The reason for this is that the background dimuons largely dominate over the nonprompt ${\mathrm {J}/\psi }$ signal.

The uncertainties in the acceptance and efficiency determination are evaluated with MC studies considering a broad range of $p_{\mathrm {T}}$ and angular spectra compatible with the pp and $\text {PbPb}$ data within their uncertainties. These variations yield an uncertainty about 0.2% (<1.7%) in $\mathrm {p}\mathrm {p}$ ($\text {PbPb}$) collisions, both for prompt and nonprompt ${\mathrm {J}/\psi }$ acceptance and efficiency. The statistical uncertainty of the weighting of the MC distributions, reflecting the impact of the limited knowledge on the kinematic distribution of ${\mathrm {J}/\psi }$ mesons on the acceptance and efficiency corrections, is used as systematic uncertainty. This uncertainty is at most 6% (11%) in $\mathrm {p}\mathrm {p}$ ($\text {PbPb}$) collisions at the largest $p_{\mathrm {T}}$ but it usually ranges from 1 to 3% in both collision systems. In addition, the systematic uncertainties in the T&P correction factors, arising from the limited data sample available and from the procedure itself, are taken into account, covering all parts of the muon efficiency: inner tracking and muon reconstruction, identification, and triggering. The dominant uncertainty in the T&P correction factors arises from muon reconstruction and ranges from 2 to 10% for both collision systems.

The global uncertainty in the pp luminosity measurement is 2.3% [45]. The number of minimum bias events corresponding to our dimuon sample in PbPb ($N_{\mathrm {MB}}$) comes from a simple event counting in the events selected by the Minimum Bias triggers, taking into account the trigger prescale. The corresponding uncertainty arises from the inefficiency of trigger and event selection, and is estimated to be 2%. Finally, the uncertainty in the $T_{\text {AA}}$ is estimated by varying the Glauber model parameters within their uncertainty and taking into account the uncertainty on the trigger and event selection efficiency, and ranges from 3 to 16% from the most central to the most peripheral events used in this analysis.

## Results

In this section, the results obtained for nonprompt ${\mathrm {J}/\psi }$ fractions, prompt and nonprompt ${\mathrm {J}/\psi }$ cross sections for each collision system, and nuclear modification factors $R_{\text {AA}}$ are presented and discussed. In addition, a derivation of the $\psi \text {(2S)}$ $R_{\text {AA}}$ is also presented and discussed. For all results plotted versus $p_{\mathrm {T}}$ or $|y |$, the abscissae of the points correspond to the centre of the respective bin, and the horizontal error bars reflect the width of the bin. The lower $p_{\mathrm {T}}$ thresholds in the different rapidity intervals reflect the detector acceptance. In the range $1.8< |y | < 2.4$ ${\mathrm {J}/\psi }$ are measured down to 3${\,\text {Ge}\text {V}/}\text {c}$, while for the bins with $|y | < 1.8$ they are measured down to 6.5${\,\text {Ge}\text {V}/}\text {c}$. When plotted as a function of centrality, the abscissae are the average $N_{\text {part}}$ values for minimum bias events within each centrality bin. The weighted average $N_{\text {part}}$ values (weighted for the number of binary nucleon-nucleon collisions) correspond in most cases to the average $N_{\text {part}}$ values for minimum bias events, with the exception of the most peripheral bin (50–100%) where $N_{\text {part}}$ changes from 22 to 43. The centrality binning used is 0–5–10–15–20–25–30–35–40–45–50–60–70–100% for the results in $|y |<2.4$, and 0–10–20–30–40–50–100% for the results differential in rapidity.

### Nonprompt ${\mathrm {J}/\psi }$ meson fractions

The nonprompt ${\mathrm {J}/\psi }$ meson fraction is defined as the proportion of measured ${\mathrm {J}/\psi }$ mesons coming from b hadron decays, corrected for acceptance and efficiency. It is presented in Fig. 2 for $\mathrm {p}\mathrm {p}$ and $\text {PbPb}$ collisions, as a function of $p_{\mathrm {T}}$ and rapidity, in the full $|y |<2.4$ and $6.5<p_{\mathrm {T}} <50{\,\text {Ge}\text {V}/}\text {c}$ range. No significant rapidity dependence is observed, while there is a strong $p_{\mathrm {T}}$ dependence, from about 20% at low $p_{\mathrm {T}}$ to 60% at high $p_{\mathrm {T}}$, reflecting the different $p_{\mathrm {T}}$ distributions of prompt and nonprompt ${\mathrm {J}/\psi }$ mesons, which highlights the necessity of separating the two contributions.

**Fig. 5:**
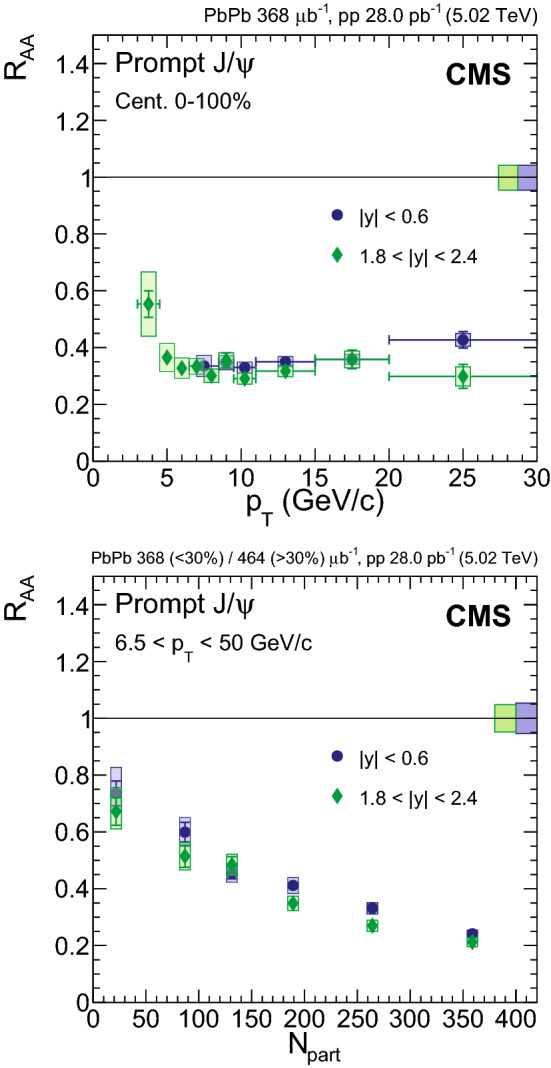
Nuclear modification factor of prompt ${\mathrm {J}/\psi }$ meson as a function of dimuon $p_{\mathrm {T}}$ (upper) and $N_{\text {part}}$ (lower), in the mid- and most forward rapidity intervals. For the results as a function of $N_{\text {part}}$ the most central bin corresponds to 0–10%, and the most peripheral one to 50–100%. The bars (boxes) represent statistical (systematic) point-by-point uncertainties. The boxes plotted at $R_{\text {AA}} =1$ indicate the size of the global relative uncertainties

**Fig. 6:**
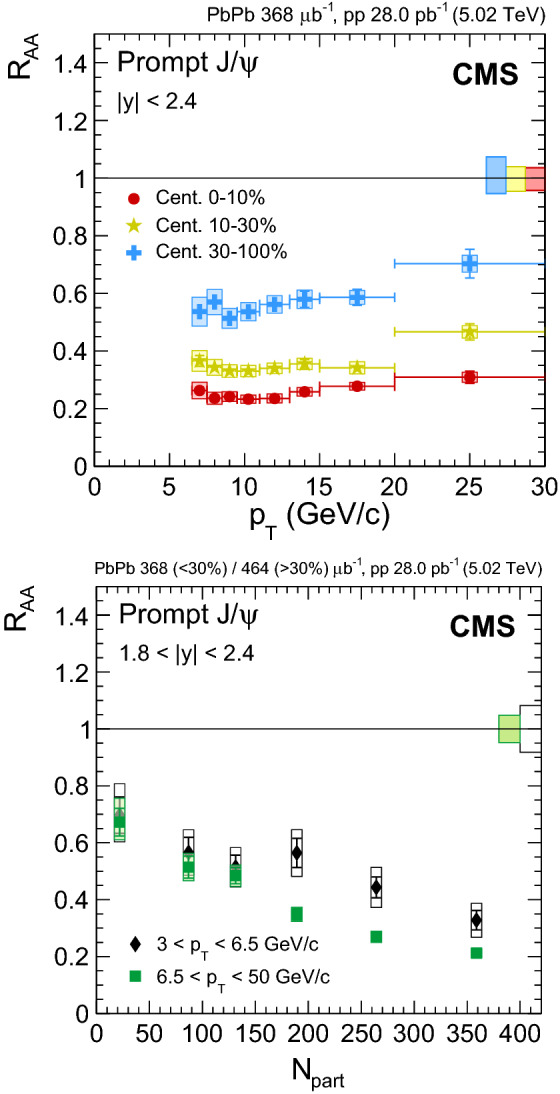
Nuclear modification factor of prompt ${\mathrm {J}/\psi }$ mesons. Upper: as a function of dimuon $p_{\mathrm {T}}$ in three centrality bins. Lower: as a function of $N_{\text {part}}$ at moderate and high $p_{\mathrm {T}}$, in the forward $1.8<|y |<2.4$ range. For the results as a function of $N_{\text {part}}$ the most central bin corresponds to 0–10%, and the most peripheral one to 50–100%. The bars (boxes) represent statistical (systematic) point-by-point uncertainties. The boxes plotted at $R_{\text {AA}} =1$ indicate the size of the global relative uncertainties

**Fig. 7:**
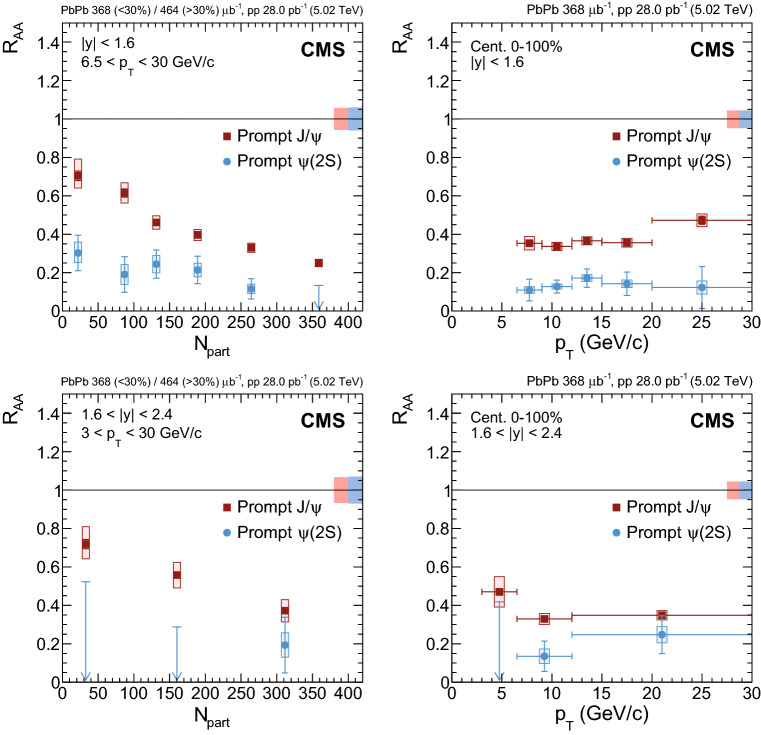
Nuclear modification factor of prompt ${\mathrm {J}/\psi }$ and $\psi \text {(2S)}$ mesons as a function of $N_{\text {part}}$ (left) and dimuon $p_{\mathrm {T}}$ (right), at central (upper, starting at $p_{\mathrm {T}} =6.5$ ${\,\text {Ge}\text {V}/}\text {c}$) and forward (lower, starting at $p_{\mathrm {T}} =3.0$ ${\,\text {Ge}\text {V}/}\text {c}$) rapidity. The vertical arrows represent 95% confidence intervals in the bins where the double ratio measurement is consistent with 0 (see text). For the results as a function of $N_{\text {part}}$ the most central bin corresponds to 0–10% (0–20%), and the most peripheral one to 50–100% (40–100%), for $|y |<1.6$ ($1.6<|y |<2.4$). The bars (boxes) represent statistical (systematic) point-by-point uncertainties. The boxes plotted at $R_{\text {AA}} = 1$ indicate the size of the global relative uncertainties

**Fig. 8:**
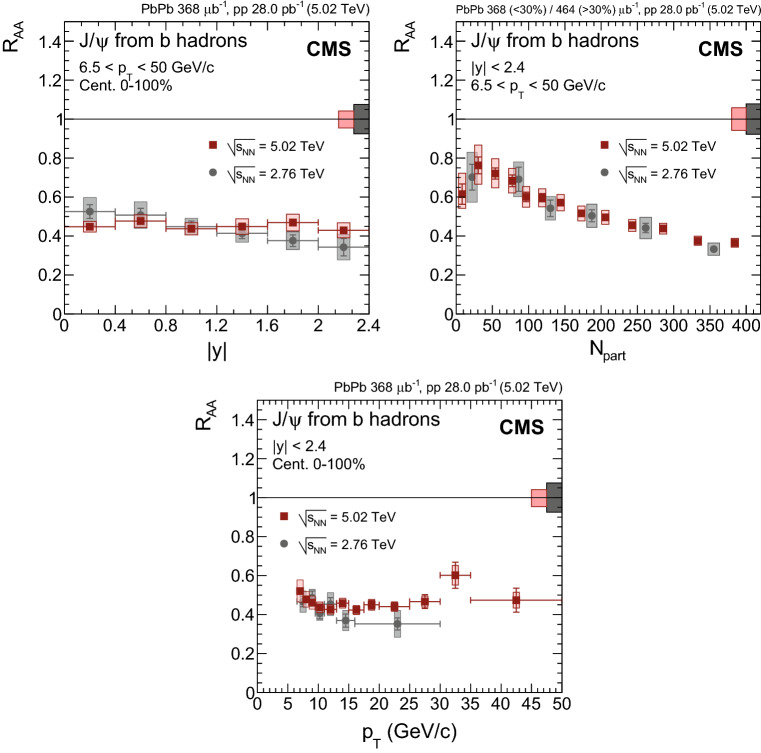
Nuclear modification factor of ${\mathrm {J}/\psi }$ mesons from b hadrons (nonprompt ${\mathrm {J}/\psi }$) as a function of dimuon rapidity (upper left), $N_{\text {part}}$ (upper right) and dimuon $p_{\mathrm {T}}$ (lower) at $\sqrt{\smash [b]{s_{_{\text {NN}}}}} = 5.02$ $\,\text {Te}\text {V}$. For the results as a function of $N_{\text {part}}$ the most central bin corresponds to 0–5%, and the most peripheral one to 70–100%. Results obtained at 2.76$\,\text {Te}\text {V}$ are overlaid for comparison [12]. The bars (boxes) represent statistical (systematic) point-by-point uncertainties. The boxes plotted at $R_{\text {AA}} =1$ indicate the size of the global relative uncertainties

### Prompt and nonprompt ${\mathrm {J}/\psi }$ meson cross sections in $\mathrm {p}\mathrm {p}$ and $\text {PbPb}$ collisions

The measurements of the prompt and nonprompt ${\mathrm {J}/\psi }$ cross sections can help to test the existing theoretical models of both quarkonium production and b hadron production. The cross sections are computed from the corrected yields,

1 $$\begin{aligned} \frac{\mathrm {d}^2 N}{\mathrm {d}p_{\mathrm {T}} \,\mathrm {d}{}y} = \frac{1}{\varDelta p_{\mathrm {T}} \, \varDelta y} \, \frac{N_{{\mathrm {J}/\psi }}}{\mathcal {A} \, \epsilon }, \end{aligned}$$

where $N_{{\mathrm {J}/\psi }}$ is the number of prompt or nonprompt ${\mathrm {J}/\psi }$ mesons, $\mathcal {A}$ is the acceptance, $\epsilon$ is the efficiency, and $\varDelta p_{\mathrm {T}}$ and $\varDelta y$ are the $p_{\mathrm {T}}$ and rapidity bin widths, respectively. To put the $\mathrm {p}\mathrm {p}$ and $\text {PbPb}$ data on a comparable scale, the corrected yields are normalised by the measured integrated luminosity for pp collisions ($\sigma = N / \mathcal {L}$), and by the product of the number of corresponding minimum bias events and the centrality-integrated nuclear overlap value for $\text {PbPb}$ collisions ($N/(N_{\mathrm {MB}}T_{\text {AA}})$). Global uncertainties (common to all measurements) arise from these normalisation factors and account for the integrated luminosity uncertainty in $\mathrm {p}\mathrm {p}$ collisions (±2.3%) and the $N_{\text {MB}}$ and $T_{\text {AA}}$ uncertainty for $\text {PbPb}$ collisions $\left( ^{+3.4\%}_{-3.9\%}\right)$, respectively.

The cross sections for the production of prompt and nonprompt ${\mathrm {J}/\psi }$ mesons that decay into two muons ($\mathcal {B} \sigma$, where $\mathcal {B}$ is the branching ratio of ${\mathrm {J}/\psi }$ to dimuons) are reported as a function of $p_{\mathrm {T}}$ and rapidity in Fig. 3.

### Prompt ${\mathrm {J}/\psi }$ meson nuclear modification factor

In order to compute the nuclear modification factor $R_{\text {AA}}$ in a given bin of centrality (cent.), the above-mentioned $\text {PbPb}$ and $\mathrm {p}\mathrm {p}$ normalised cross sections are divided in the following way:

$$\begin{aligned} R_{\text {AA}}= & {} \frac{N^{\text {PbPb}}_{{\mathrm {J}/\psi }} (\text {cent.}) }{N^{{\mathrm {p}\mathrm {p}}}_{{\mathrm {J}/\psi }}} \times \frac{\mathcal {A}^{{\mathrm {p}\mathrm {p}}} \times \epsilon ^{{\mathrm {p}\mathrm {p}}}}{\mathcal {A}^{\text {PbPb}} \, \epsilon ^{\text {PbPb}} (\text {cent.}) }\\&\times \frac{\mathcal {L}^{{\mathrm {p}\mathrm {p}}}}{N_{\text {MB}} \, \langle T_{\text {AA}} \rangle \, (\text {cent. fraction})}, \end{aligned}$$

where the centrality fraction is the fraction of the inclusive inelastic cross section probed in the analysis bin. Global uncertainties (indicated as boxes in the plots at $R_{\text {AA}} =1$) arise from the full $\mathrm {p}\mathrm {p}$ statistical and systematic uncertainties and the $\text {PbPb}$ $N_{\text {MB}}$ uncertainty when binning as a function of the centrality; and from the integrated luminosity of the $\mathrm {p}\mathrm {p}$ data, and the $N_{\text {MB}}$ and $T_{\text {AA}}$ uncertainties of the $\text {PbPb}$ data, when binning as a function of rapidity or $p_{\mathrm {T}}$.

In Fig. 4, the $R_{\text {AA}}$ of prompt ${\mathrm {J}/\psi }$ mesons as a function of rapidity, $N_{\text {part}}$ and $p_{\mathrm {T}}$ are shown, integrating in each case over the other two non-plotted variables. The results are compared to those obtained at $\sqrt{\smash [b]{s_{_{\text {NN}}}}} = 2.76$ $\,\text {Te}\text {V}$  [12], and they are found to be in good overall agreement. No strong rapidity dependence of the suppression is observed. As a function of centrality, the $R_{\text {AA}}$ is suppressed even for the most peripheral bin (70–100%), with the suppression slowly increasing with $N_{\text {part}}$. The $R_{\text {AA}}$ value for the most central events (0–5%) is measured for $6.5<p_{\mathrm {T}} <50$ ${\,\text {Ge}\text {V}/}\text {c}$ and $|y |<2.4$ to be $0.219 \pm 0.005\,\text {(stat)} \pm 0.013\,\text {(syst)}$. As a function of $p_{\mathrm {T}}$ the $R_{\text {AA}}$ is approximately constant in the range of 5–20${\,\text {Ge}\text {V}/}\text {c}$, but an indication of less suppression at higher $p_{\mathrm {T}}$ is seen for the first time in quarkonia. Charged hadrons, for which the suppression is usually attributed to parton energy loss [16, 46], show a similar increase in $R_{\text {AA}}$ at high $p_{\mathrm {T}}$ for $\text {PbPb}$ collisions at $\sqrt{\smash [b]{s_{_{\text {NN}}}}} = 5.02$ $\,\text {Te}\text {V}$  [27].

Double-differential studies are also performed. Figure 5 shows the $p_{\mathrm {T}}$ (upper) and centrality (lower) dependence of prompt ${\mathrm {J}/\psi }$ $R_{\text {AA}}$ measured in the mid- and most forward rapidity intervals. A similar suppression pattern is observed for both rapidities. Figure 6 (upper) shows the dependence of $R_{\text {AA}}$ as a function of $p_{\mathrm {T}}$, for three centrality intervals. Although the mean level of suppression strongly depends on the sampled centrality range, the general trend of the $p_{\mathrm {T}}$ dependence appears similar in all three centrality ranges, including the increase of $R_{\text {AA}}$ at high $p_{\mathrm {T}}$. Finally, Fig. 6 (lower) considers the rapidity interval $1.8<|y |<2.4$, where the acceptance goes down at lower $p_{\mathrm {T}}$. The suppression is found to be similar in peripheral events at moderate ($3<p_{\mathrm {T}} <6.5$ ${\,\text {Ge}\text {V}/}\text {c}$) and high ($6.5<p_{\mathrm {T}} <50$ ${\,\text {Ge}\text {V}/}\text {c}$) transverse momentum ranges, but it is weaker for lower $p_{\mathrm {T}}$ in the most central region. This is also reflected in the first bin of the most forward measurement in Fig. 5 (upper). A similarly reduced suppression at low $p_{\mathrm {T}}$ is observed by the ALICE Collaboration, which is attributed to a regeneration contribution [9, 10].

### Prompt $\psi \text {(2S)}$ meson nuclear modification factor

Having measured the prompt ${\mathrm {J}/\psi }$ $R_{\text {AA}}$, one can derive that of the $\psi \text {(2S)}$ meson by multiplying it by the double ratio $(N_{\psi \text {(2S)}}/N_{{\mathrm {J}/\psi }})_{\text {PbPb}}/ (N_{\psi \text {(2S)}}/N_{{\mathrm {J}/\psi }})_{{\mathrm {p}\mathrm {p}}}$ of the relative modification of the prompt $\psi \text {(2S)}$ and ${\mathrm {J}/\psi }$ meson yields from $\mathrm {p}\mathrm {p}$ to $\text {PbPb}$ collisions published in Ref. [47]. Since the $\psi \text {(2S)}$ yield suffers from lower statistics, the current ${\mathrm {J}/\psi }$ analysis is repeated using the wider bins of Ref. [47]. The centrality binning used is 0–10–20–30–40–50–100% for the results in $|y |<1.6$, and 0–20–40–100% for the results in $1.6<|y |<2.4$. Since the statistical uncertainty in the $\psi \text {(2S)}$ largely dominates, the ${\mathrm {J}/\psi }$ uncertainties are propagated by considering them to be uncorrelated to the double ratio uncertainties.

The results are presented in Fig. 7 as a function of dimuon $p_{\mathrm {T}}$ and $N_{\text {part}}$, in two rapidity ranges of different $p_{\mathrm {T}}$ reach. In the bins where the double ratio is consistent with 0, 95% CL intervals on the prompt $\psi \text {(2S)}$ $R_{\text {AA}}$ are derived using the Feldman–Cousins procedure [48]. The procedure to obtain the CL intervals is the same as in the double ratio measurement, incorporating the ${\mathrm {J}/\psi }$ $R_{\text {AA}}$ statistical and systematic uncertainties as a nuisance parameter. It can be observed that the $\psi \text {(2S)}$ meson production is more suppressed than that of ${\mathrm {J}/\psi }$ mesons, in the entire measured range. The $\psi \text {(2S)}$ meson $R_{\text {AA}}$ shows no clear dependence of the suppression with $p_{\mathrm {T}}$, and hints of an increasing suppression with collision centrality. These results show that the $\psi \text {(2S)}$ mesons are more strongly affected by the medium created in $\text {PbPb}$ collisions than the ${\mathrm {J}/\psi }$ mesons.

**Fig. 9:**
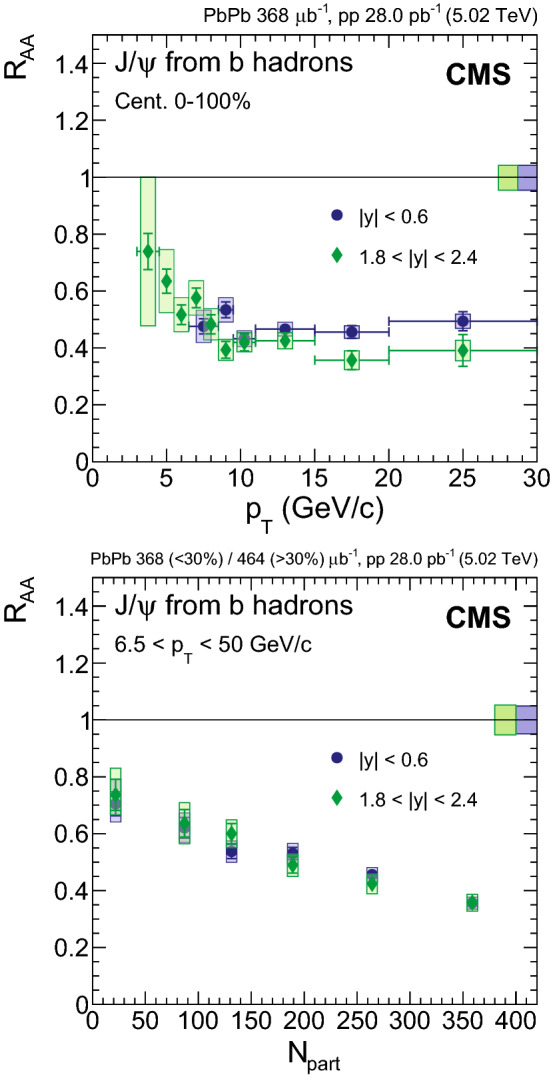
Nuclear modification factor of ${\mathrm {J}/\psi }$ mesons from b hadrons (nonprompt ${\mathrm {J}/\psi }$) as a function of dimuon $p_{\mathrm {T}}$ (upper) and $N_{\text {part}}$ (lower) and in the mid- and most forward rapidity intervals. For the results as a function of $N_{\text {part}}$ the most central bin corresponds to 0–10%, and the most peripheral one to 50–100%. The bars (boxes) represent statistical (systematic) point-by-point uncertainties. The boxes plotted at $R_{\text {AA}} =1$ indicate the size of the global relative uncertainties

**Fig. 10:**
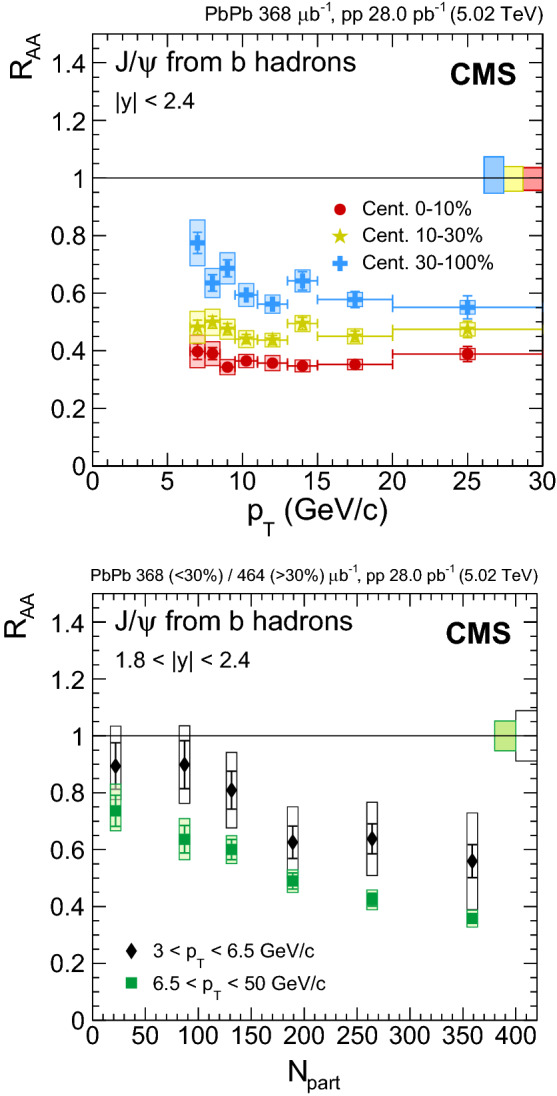
Nuclear modification factor of ${\mathrm {J}/\psi }$ mesons from b hadrons (nonprompt ${\mathrm {J}/\psi }$). Upper: as a function of dimuon $p_{\mathrm {T}}$ in three centrality bins. Lower: as a function of $N_{\text {part}}$ at moderate and high $p_{\mathrm {T}}$, in the forward $1.8<|y |<2.4$ range. For the results as a function of $N_{\text {part}}$ the most central bin corresponds to 0–10%, and the most peripheral one to 50–100%. The bars (boxes) represent statistical (systematic) point-by-point uncertainties. The boxes plotted at $R_{\text {AA}} =1$ indicate the size of the global relative uncertainties

### Nonprompt ${\mathrm {J}/\psi }$ meson nuclear modification factor

The procedure applied to derive the prompt ${\mathrm {J}/\psi }$ meson $R_{\text {AA}}$ is applied to the nonprompt component. In Fig. 8, the $R_{\text {AA}}$ of nonprompt ${\mathrm {J}/\psi }$ as a function of rapidity, centrality and $p_{\mathrm {T}}$ are shown, integrating in each case over the other two non-plotted variables. The results are compared to those obtained at $\sqrt{\smash [b]{s_{_{\text {NN}}}}} = 2.76$ $\,\text {Te}\text {V}$  [12]. A good overall agreement is found, although no rapidity dependence is observed in the present analysis, while the suppression was slowly increasing towards forward rapidities in the lower-energy measurement. A steady increase of the suppression is observed with increasing centrality of the collision. The $R_{\text {AA}}$ for the most central events (0–5%) measured for $6.5<p_{\mathrm {T}} <50$ ${\,\text {Ge}\text {V}/}\text {c}$ and $|y |<2.4$ is $0.365\pm 0.009\,\text {(stat)} \pm 0.022\,\text {(syst)}$.

As for the prompt production case, double-differential studies are also performed. Figure 9 shows the $p_{\mathrm {T}}$ (upper) and centrality (lower) dependence of nonprompt ${\mathrm {J}/\psi }$ meson $R_{\text {AA}}$ measured in the mid- and most forward rapidity intervals. No strong rapidity dependence is observed, and a hint of a smaller suppression at low $p_{\mathrm {T}}$ is seen in the $1.8<|y |<2.4$ range. Figure 10 (upper) shows the dependence of $R_{\text {AA}}$ as a function of $p_{\mathrm {T}}$, for three centrality ranges. While the nonprompt ${\mathrm {J}/\psi }$ meson $R_{\text {AA}}$ does not seem to depend on rapidity, the data indicates a larger $p_{\mathrm {T}}$ dependence in peripheral events. Finally, Fig. 10 (lower) shows, for $1.8<|y |<2.4$, $R_{\text {AA}}$ as a function of $N_{\text {part}}$, for two $p_{\mathrm {T}}$ intervals. Hints of a stronger suppression are seen for $p_{\mathrm {T}} >6.5{\,\text {Ge}\text {V}/}\text {c}$ at all centralities.

## Conclusions

Prompt and nonprompt ${\mathrm {J}/\psi }$ meson production has been studied in $\mathrm {p}\mathrm {p}$ and $\text {PbPb}$ collisions at $\sqrt{\smash [b]{s_{_{\text {NN}}}}} = 5.02\,\text {Te}\text {V}$, as a function of rapidity, transverse momentum ($p_{\mathrm {T}}$), and collision centrality, in different kinematic and centrality ranges. Three observables were measured: nonprompt ${\mathrm {J}/\psi }$ fractions, prompt and nonprompt ${\mathrm {J}/\psi }$ cross sections for each collision system, and nuclear modification factors $R_{\text {AA}}$. The $R_{\text {AA}}$ results show a strong centrality dependence, with an increasing suppression for increasing centrality. For both prompt and nonprompt ${\mathrm {J}/\psi }$ mesons no significant dependence on rapidity is observed. An indication of less suppression in the lowest $p_{\mathrm {T}}$ range at forward rapidity is seen for both ${\mathrm {J}/\psi }$ components. Double-differential measurements show the same trend, and also suggest a stronger $p_{\mathrm {T}}$ dependence in peripheral events. An indication of less suppression of the prompt ${\mathrm {J}/\psi }$ meson at high $p_{\mathrm {T}}$ is seen with respect to that observed at intermediate $p_{\mathrm {T}}$. The measurements are consistent with previous results at $\sqrt{\smash [b]{s_{_{\text {NN}}}}} = 2.76\,\text {Te}\text {V}$.

Combined with previous results for the double ratio $(N_{\psi \text {(2S)}}/N_{{\mathrm {J}/\psi }})_{\text {PbPb}}/ (N_{\psi \text {(2S)}}/N_{{\mathrm {J}/\psi }})_{{\mathrm {p}\mathrm {p}}}$, the current $R_{\text {AA}}$ values for ${\mathrm {J}/\psi }$ mesons are used to derive the prompt $\psi \text {(2S)}$ meson $R_{\text {AA}}$ in $\text {PbPb}$ collisions at $\sqrt{\smash [b]{s_{_{\text {NN}}}}} = 5.02\,\text {Te}\text {V}$, as a function of $p_{\mathrm {T}}$ and collision centrality, in two different rapidity ranges. The results show that the $\psi \text {(2S)}$ is more suppressed than the ${\mathrm {J}/\psi }$ meson for all the kinematical ranges studied. No $p_{\mathrm {T}}$ dependence is observed within the current uncertainties. Hints of an increase in suppression with increasing collision centrality are also observed.

## References

[1] Matsui T, Satz H (1986). $$\text{J}/\psi $$ suppression by quark-gluon plasma formation. Phys. Lett. B.

[2] Digal S, Petreczky P, Satz H (2001). Quarkonium feed down and sequential suppression. Phys. Rev. D.

[3] NA50 Collaboration, A new measurement of $$\text{ J }/\psi $$ suppression in $$\text{ PbPb }$$ collisions at $$158 \text{ GeV }$$ per nucleon. Eur. Phys. J. C **39**, 335 (2005). 10.1140/epjc/s2004-02107-9. arXiv:hep-ex/0412036

[4] PHENIX Collaboration, $$\text{ J }/\psi $$ production versus centrality, transverse momentum, and rapidity in $$\text{ AuAu }$$ collisions at $$\sqrt{s\_{{\rm nn}}} = 200 \text{ GeV }$$. Phys. Rev. Lett. **98**, 232301 (2007). 10.1103/PhysRevLett.98.232301. arXiv:nucl-ex/0611020 10.1103/PhysRevLett.98.232301 17677901

[5] CMS Collaboration, Observation of sequential $$\varUpsilon $$ suppression in $$\text{ PbPb }$$ collisions. Phys. Rev. Lett. **109**, 222301 (2012). 10.1103/PhysRevLett.109.222301. arXiv:1208.2826 10.1103/PhysRevLett.120.199903 29799238

[6] ALICE Collaboration, Suppression of $$\varUpsilon (1S)$$ at forward rapidity in Pb–Pb collisions at $$\sqrt{s\_{{\rm nn}}} = 2.76 \text{ TeV }$$. Phys. Lett. B **738**, 361 (2014). 10.1016/j.physletb.2014.10.001. arXiv:1405.4493

[7] Braun-Munzinger P, Stachel J (2000). (Non)thermal aspects of charmonium production and a new look at $$\text{ J }/\psi $$ suppression. Phys. Lett. B.

[8] Thews RL, Schroedter M, Rafelski J (2001). Enhanced $$\text{ J }/\psi $$ production in deconfined quark matter. Phys. Rev. C.

[9] ALICE Collaboration, $$\text{ J }/\psi $$ suppression at forward rapidity in $$\text{ PbPb }$$ collisions at $$\sqrt{s\_{{\rm nn}}} = 2.76 \text{ TeV }$$. Phys. Rev. Lett. **109**, 072301 (2012). 10.1103/PhysRevLett.109.072301. arXiv:1202.1383

[10] ALICE Collaboration, $$\text{ J }/\psi $$ suppression at forward rapidity in $$\text{ PbPb }$$ collisions at $$\sqrt{s\_{{\rm nn}}} = 5.02 \text{ TeV }$$. Phys. Lett. B **766**, 212 (2017). 10.1016/j.physletb.2016.12.064. arXiv:1606.08197

[11] PHENIX Collaboration, $$\text{ J }/\psi $$ suppression at forward rapidity in $$\text{ AuAu }$$ collisions at $$\sqrt{s\_{{\rm nn}}} = 200 \text{ GeV }$$. Phys. Rev. C **84**, 054912 (2011). 10.1103/PhysRevC.84.054912. arXiv:1103.6269

[12] CMS Collaboration, Suppression and azimuthal anisotropy of prompt and nonprompt $${\rm J}/\psi $$ production in PbPb collisions at $$\sqrt{s\_{\text{ NN }}} =2.76\,{{\rm TeV}}$$. Eur. Phys. J. C **77**, 252 (2017). 10.1140/epjc/s10052-017-4781-1. arXiv:1610.00613 10.1140/epjc/s10052-017-4781-1 PMC5409806 28515669

[13] Strickland M (2011). Thermal $${\varUpsilon }_{1S}$$ and $$\chi_{\rm b1}$$ suppression in $$\sqrt{s_{{\rm nn}}} =2.76$$ TeV Pb-Pb collisions at the LHC. Phys. Rev. Lett..

[14] Du X, Rapp R (2015). Sequential regeneration of charmonia in heavy-ion collisions. Nucl. Phys. A.

[15] Spousta M (2017). On similarity of jet quenching and charmonia suppression. Phys. Lett. B.

[16] Arleo F (2017). Quenching of hadron spectra in heavy ion collisions at the LHC. Phys. Rev. Lett..

[17] LHCb Collaboration, Measurement of $$\text{ J }/\psi $$ production in $$\rm pp$$ collisions at $$\sqrt{s} = 7 \text{ TeV }$$. Eur. Phys. J. C **71**, 1645 (2011). 10.1140/epjc/s10052-011-1645-y. arXiv:1103.0423

[18] CMS Collaboration, Prompt and non-prompt $$\text{ J }/\psi $$ production in $${{\rm pp}}$$ collisions at $$\sqrt{s} = 7 \text{ TeV }$$. Eur. Phys. J. C **71**, 1575 (2011). 10.1140/epjc/s10052-011-1575-8. arXiv:1011.4193

[19] ATLAS Collaboration, Measurement of the differential cross-sections of inclusive, prompt and non-prompt $$\text{ J }/\psi $$ production in $${{\rm pp}}$$ collisions at $$\sqrt{s} = 7\text{ TeV }$$. Nucl. Phys. B **850**, 387 (2011). 10.1016/j.nuclphysb.2011.05.015. arXiv:1104.3038

[20] CMS Collaboration, The CMS trigger system. JINST **12**, P01020 (2017). 10.1088/1748-0221/12/01/P01020. arXiv:1609.02366

[21] CMS Collaboration, The CMS experiment at the CERN LHC. JINST **3**, S08004 (2008). 10.1088/1748-0221/3/08/S08004

[22] CMS Collaboration, Description and performance of track and primary-vertex reconstruction with the CMS tracker. JINST **9**, P10009 (2014). 10.1088/1748-0221/9/10/P10009

[23] Cacciari M, Salam GP, Soyez G (2008). The anti-$$k_t$$ jet clustering algorithm. JHEP.

[24] Cacciari M, Salam GP, Soyez G (2012). FastJet user manual. Eur. Phys. J. C.

[25] CMS Collaboration, Transverse momentum and pseudorapidity distributions of charged hadrons in pp collisions at $$\sqrt{s} = 0.9$$ and 2.36 TeV. JHEP **02**, 041 (2010). 10.1007/JHEP02(2010)041. arXiv:1002.0621 10.1103/PhysRevLett.105.022002 20867699

[26] CMS Collaboration, Dependence on pseudorapidity and centrality of charged hadron production in $$\text{ PbPb }$$ collisions at $$\sqrt{s\_{{\rm nn}}} = 2.76 \text{ TeV }$$. JHEP **08**, 141 (2011). 10.1007/JHEP08(2011)141. arXiv:1107.4800

[27] CMS Collaboration, Charged-particle nuclear modification factors in PbPb and pPb collisions at $$\sqrt{s\_{{\rm nn}}} =5.02 \text{ TeV }$$. JHEP **04**, 039 (2017). 10.1007/JHEP04(2017)039. arXiv:1611.01664

[28] Miller ML, Reygers K, Sanders SJ, Steinberg P (2007). Glauber modeling in high-energy nuclear collisions. Ann. Rev. Nucl. Part. Sci..

[29] Sjöstrand T, Mrenna S, Skands P (2008). A brief introduction to PYTHIA 8.1. Comput. Phys. Commun..

[30] Lange DJ (2001). The EvtGen particle decay simulation package. Nucl. Instrum. Methods A.

[31] Lokhtin IP, Snigirev AM (2006). A model of jet quenching in ultrarelativistic heavy ion collisions and high-$$p_{\rm T}$$ hadron spectra at RHIC. Eur. Phys. J. C.

[32] GEANT Collaboration, GEANT4 — a simulation toolkit. Nucl. Instrum. Methods A **506**, 250 (2003). 10.1016/S0168-9002(03)01368-8

[33] ALICE Collaboration, $$\text{ J }/\psi $$ polarization in $${{\rm pp}}$$ collisions at $$\sqrt{s} = 7 \text{ TeV }$$. Phys. Rev. Lett. **108**, 082001 (2011). 10.1103/PhysRevLett.108.082001. arXiv:1111.1630

[34] CMS Collaboration, Measurement of the prompt $$\text{ J }/\psi $$ and $$\psi \text{(2S) }$$ polarizations in $${{\rm pp}}$$ collisions at $$\sqrt{s} = 7 \text{ TeV }$$. Phys. Lett. B **727**, 381 (2013). 10.1016/j.physletb.2013.10.055. arXiv:1307.6070

[35] LHCb Collaboration, Measurement of $$\text{ J }/\psi $$ polarization in $${{\rm pp}}$$ collisions at $$\sqrt{s} = 7 \text{ TeV }$$. Eur. Phys. J. C **73**, 2631 (2013). 10.1140/epjc/s10052-013-2631-3. arXiv:1307.6379 10.1140/epjc/s10052-013-2631-3 PMC4370894 25814848

[36] CMS Collaboration, Suppression of non-prompt $$\text{ J }/\psi $$, prompt $$\text{ J }/\psi $$, and $$\varUpsilon {\rm (1S)}$$ in PbPb collisions at $$\sqrt{s\_{{\rm nn}}} = 2.76 \text{ TeV }$$. JHEP **05**, 063 (2012). 10.1007/JHEP05(2012)063. arXiv:1201.5069

[37] CMS Collaboration, Measurement of quarkonium production cross sections in pp collisions at $$\sqrt{s}=$$ 13 TeV. Phys. Lett. B **780**, 251–272 (2018). 10.1016/j.physletb.2018.02.033. arXiv:1710.11002

[38] CMS Collaboration, $$\varUpsilon ({{\rm nS}})$$ polarizations versus particle multiplicity in pp collisions at $$\sqrt{s} =$$ 7 TeV. Phys. Lett. B **761**, 31–52 (2016). 10.1016/j.physletb.2016.07.065. arXiv:1603.02913

[39] CMS Collaboration, Performance of CMS muon reconstruction in pp collision events at $$\sqrt{s} = 7 \text{ TeV }$$. JINST **7**, P10002 (2012). 10.1088/1748-0221/7/10/P10002. arXiv:1206.4071

[40] ALEPH Collaboration, Measurement of the $$\text{ anti-B }^{0}$$ and $$\text{ B }^{-}$$ meson lifetimes. Phys. Lett. B **307**, 194 (1993). 10.1016/0370-2693(93)90211-Y (errata: 10.1016/0370-2693(94)90054-X)

[41] Particle Data Group, C. Patrignani et al., Review of particle physics. Chin. Phys. C **40**, 100001 (2016). 10.1088/1674-1137/40/10/100001

[42] M.J. Oreglia, A study of the reactions $$\psi ^\prime \rightarrow \gamma \gamma \psi $$. PhD thesis, Stanford University (1980) (SLAC report SLAC-R-236, see Appendix D)

[43] Pivk M, Le Diberder FR (2005). sPlot: a statistical tool to unfold data distributions. Nucl. Instrum. Methods A.

[44] CMS Collaboration, Measurements of inclusive $$W$$ and $$Z$$ cross sections in $${{\rm pp}}$$ collisions at $$\sqrt{s}=7$$ TeV. JHEP **01**, 080 (2011). 10.1007/JHEP01(2011)080. arXiv:1012.2466

[45] CMS Collaboration, CMS luminosity calibration for the pp reference run at $$\sqrt{s}=5.02\,{{\rm TeV}}$$. CMS Physics Analysis Summary CMS-PAS-LUM-16-001 (2016)

[46] D. d’Enterria, Jet quenching, in *Springer Materials—The Landolt–Börnstein Database*, vol. 23, ed. by R. Stock. Relativistic Heavy Ion Physics (Springer, New York, 2010), p. 9. 10.1007/978-3-642-01539-7_16. arXiv:0902.2011

[47] CMS Collaboration, Relative modification of prompt $$\psi $$(2S) and $$\text{ J }/\psi $$ yields from $$pp$$ to PbPb collisions at $$\sqrt{s\_{{\rm nn}}} = 5.02 \text{ TeV }$$. Phys. Rev. Lett. **118**, 162301 (2017). 10.1103/PhysRevLett.118.162301. arXiv:1611.01438 10.1103/PhysRevLett.118.162301 28474955

[48] Feldman GJ, Cousins RD (1998). A unified approach to the classical statistical analysis of small signals. Phys. Rev. D.

